# Val143 of human ribonuclease H2 is not critical for, but plays a role in determining catalytic activity and substrate specificity

**DOI:** 10.1371/journal.pone.0228774

**Published:** 2020-02-18

**Authors:** Misato Baba, Kenji Kojima, Takuto Nishimura, Takuya Sugiura, Teisuke Takita, Ryo Uehara, Robert J. Crouch, Kiyoshi Yasukawa

**Affiliations:** 1 Division of Food Science and Biotechnology, Graduate School of Agriculture, Kyoto University, Kyoto, Japan; 2 Section on Formation of RNA, Intramural Research Program, *Eunice Kennedy Shriver* National Institute of Child Health and Human Development, National Institutes of Health, Bethesda, MD, United States of America; 3 Ritsumeikan Global Innovation Research Organization, Ritsumeikan University, Noji-higashi, Shiga, Japan; Universität Stuttgart, GERMANY

## Abstract

Ribonuclease H2 (RNase H2) exhibits both single ribonucleotide excision activity (activity A) and RNA strand degrading activity (activity B). Val143 of human RNase H2 is located at the active site and is conserved in eukaryotic RNase H2. In this study, we explored the role of Val143 in catalytic activity and substrate specificity. Nineteen single variants at amino acid position 143 were expressed in *E*. *coli*, and all variants except for V143C and V143M were purified from the cells. When the activity of the wild-type human RNase H2 (WT) was set as 100%, the relative activities A and B of the 17 variants were in the range of 0.05–130 and 0.02–42%, respectively. When the ratio of the relative activity A to the relative activity B of WT was set as 1, the ratios of the 17 variants were in the range of 0.2–5.7. This indicates that valine is optimal for balancing the two activities. The ratios for V143Y and V143W were relatively high (5.6 and 5.5, respectively), suggesting that the bulky residues like tyrosine and tryptophan at position 143 caused steric hindrance with the 2’-OH of the sugar moiety of the ribonucleotide at the 5’ side of the scissile phosphodiester bond. The ratio for V143Q was relatively low (0.2). These results suggested that Val143 is not critical for, but plays a role in determining catalytic activity and substrate specificity.

## Introduction

Ribonuclease H (RNase H) [EC 3.1.26.4] is an enzyme that specifically degrades RNA from RNA/DNA hybrids. RNase H is divided into two groups, type 1 and type 2. The former requires at least four consecutive ribonucleotides incorporated into DNA duplex for the degradation of RNA, while the latter requires only single ribonucleotide [1–3]. Thus, type 1 enzymes exhibit the RNA strand degrading activity but lack the single ribonucleotide excision activity, while type 2 enzymes exhibit both activities. Type 1 enzymes are designated RNase HI in prokaryotes and RNase H1 in eukaryotes. Type 2 enzymes are designated RNase HII in prokaryotes and RNase H2 in eukaryotes. RNase HII is a monomer, while RNase H2 is a heterotrimer consisting of one catalytic subunit (A) (299 amino acids in human) and two accessory subunits (B and C) (312 and 164 amino acids, respectively, in human) [4–7].

DNA polymerases incorporate a single ribonucleotide every few thousand base pairs in the mouse genome [8]. Such ribonucleotides can cause double strand DNA breaks. RNase H2 is involved in the removal of such ribonucleotides, playing an important role in genome stability. Consistent with this, RNase H2 A [9], B, or C [8, 10] subunit knock-out mice were reported to be embryonic lethal. On the other hand, cells can grow in the absence of RNase H2 activity *in vitro* [8, 11–15] and *in vivo* [16, 17]

Seven crystal structures of RNases HII and RNases H2 are currently available [18]. In mouse [19] and human [6, 7] RNases H2, the C subunit is flanked by the A and B subunits, and the N-terminal domain of the B subunit and the entire C subunit are intimately interwoven to form the triple β-barrel fold, which interacts with the C-terminal domain of the A subunit. The active site in the A subunit has a conserved GRG (Gly37, Arg38, and Gly39 in human), DEDD (Asp34, Glu35, Asp141, and Asp169 in human) and DSK (Asp67, Ser68, and Lys69 in human) motifs. Residues in the DEDD motif coordinate metal ions. The GRG motif- and DSK motif-containing loops are located close to the active site. We previously examined pH and temperature dependences of human RNase H2 activity and suggested that the ionizable groups responsible for acidic p*K*_e_ may be two of the residues in the DEDD motif, and that for alkaline p*K*_e_ may be Lys69 of the DSK motif [20].

The structure of the complex of *Thermotoga maritima* RNase HII and a hybrid consisting of DNA_5_-RNA_1_-DNA_6_ and DNA_12_ revealed that the hydroxyl group of the side chain of Tyr163 is located in the proximity with the 2’-OH of the sugar moiety of the ribonucleotide at the 3’ side of the scissile phosphodiester bond of the substrate [21]. This Tyr residue is conserved in RNase HII and RNase H2. In yeast RNase H2, the counterpart of this tyrosine residue is Tyr219. The yeast RNase H2 double mutant P45D/Y219A lacked the single ribonucleotide excision activity but retained the RNA strand degrading activity [22]. These results suggested that this tyrosine residue is critical for substrate specificity.

**Fig 1:**
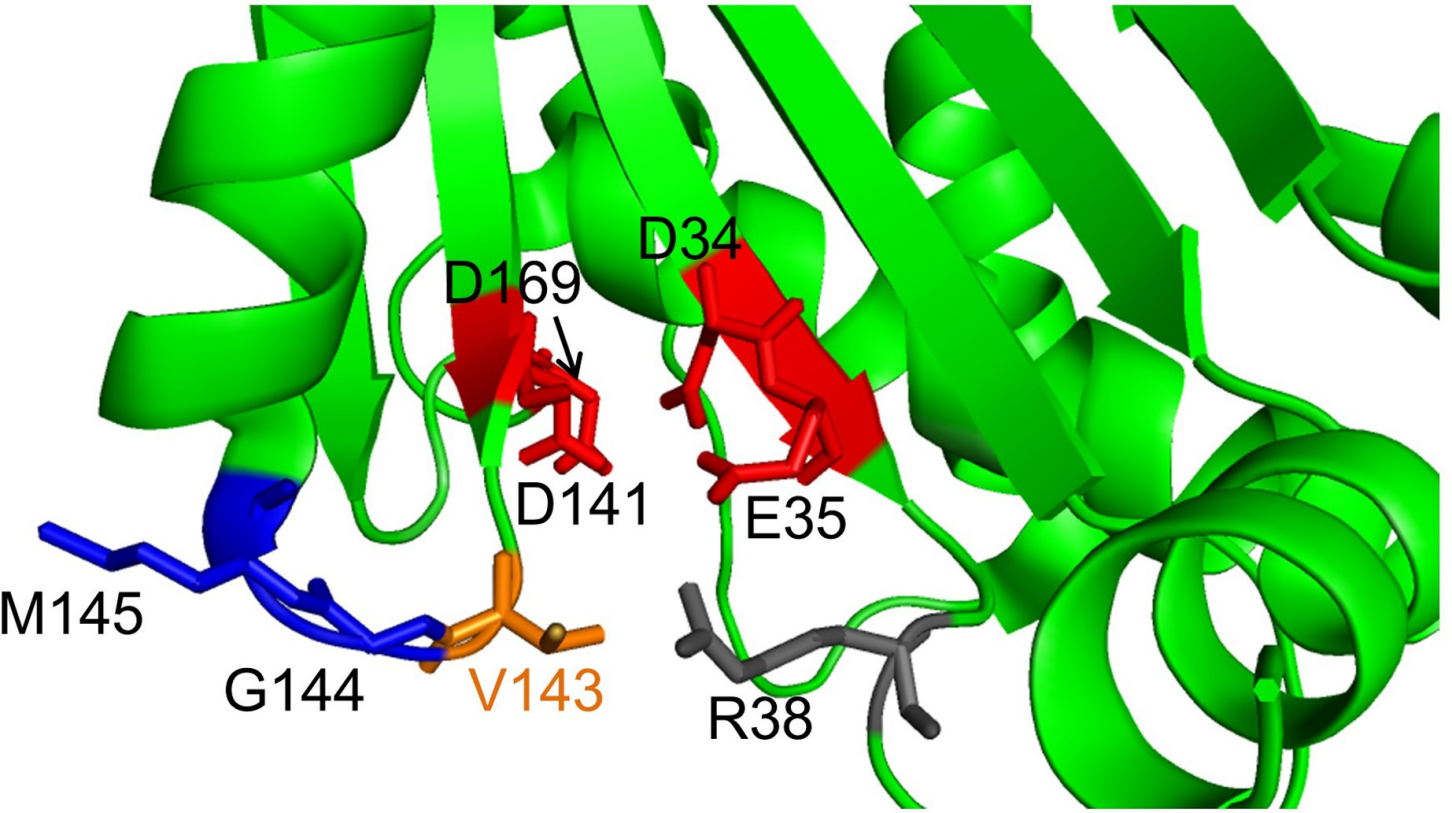
Close-up view of the active site of human RNase H2. The active site of human RNase H2 (PDB accession code 3PUF) [7] is shown. Val143, Asp169, and Arg38 are shown in orange, red, and gray, respectively, and Gly144 and Met145 are shown in blue.

**Fig 2:**
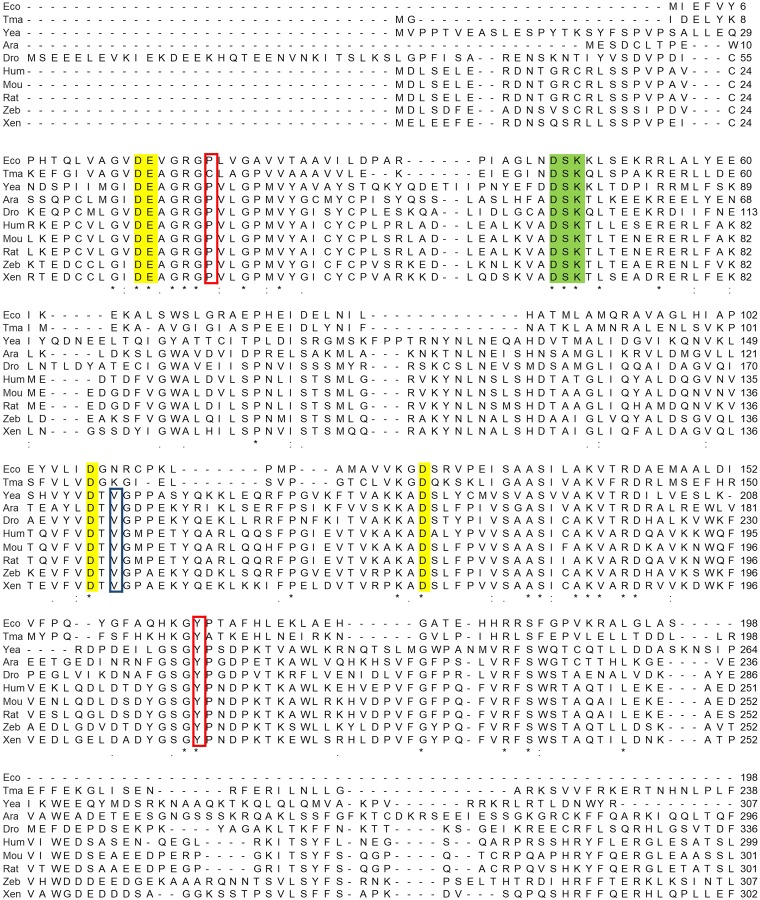
Amino acid sequences of RNases H2. A multiple sequence alignment was created using Clustal Omega. Eco, *Escherichia coli* (NP_414725.1); Tma, *Thermotoga maritima* (NP_228723.1); Yea, *Saccharomyces cerevisiae* (NP_014327.1); Ara, *Arabidopsis lyrata subsp*. *lyrata* (XP_002880652.1); Dro, *Drosophila willistoni* (XP_002074967.1); Hum, *Homo sapiens* (NP_006388.2); Mou, *Mus musculus* (NP_081463.1); Rat, *Rattus norvegicus* (NP_001013252.1); Zeb, *Danio rerio* (NP_956520.2); and Xen, *Xenopus laevis* (NP_001180335.1). Residues in the DEDD motif are highlighted in yellow, and those in the DSK motif are in green. The Pro40 and Tyr210 in human RNase H2 and their counterparts are boxed in red, and Val143 of human RNase H2 and its counterparts are boxed in blue.

Unlike the tyrosine residue mentioned above, little is known about a role of other conserved amino acid residues in the active site of RNase H2 in activity and stability. The active-site residue Val143 (Fig 1) is conserved in eukaryotic RNase H2 but not in prokaryotic RNase HII (Fig 2). RNase H2 cleaves the 5’ side of a RNA-DNA junction and the middle of an RNA stretch in an RNA/DNA hybrid, while RNase HII cleaves only the 5’ side of a RNA-DNA junction [18]. In this study, to explore the role of Val143 in the RNA strand degrading and single ribonucleotide excision activities, we performed saturation mutagenesis of this residue and analyzed variants. The results have revealed that Val143 is important to, but not critical for, catalytic activity and substrate specificity.

## Materials and methods

### Construction of plasmids

pET15b-hH2ABC [5], which was the pET-15b(+) plasmid (Merck Bioscience, Tokyo, Japan) harboring the gene encoding A, B, and C subunits of human RNase H2 with a N-terminal (His)_6_ tag at each subunit, was used as an expression plasmid of the wild-type human RNase H2 (WT). Expression plasmids of variants were constructed by site-directed mutagenesis using the pET15b-hH2ABC as a template, *E*. *coli* BL21(DE3) as a host, and the oligonucleotides listed in S1 Table as a primer.

### Expression and purification of RNase H2

WT and variants were prepared as described previously [20, 23]. Briefly, human RNase H2 was expressed in the soluble fraction of the BL21(DE3) transformants, from which active enzymes were purified by an affinity column chromatography with a HiTrap Heparin HP column (GE Healthcare, Buckinghamshire, UK) and a HisTrap HP column (GE Healthcare) followed by a gel filtration column chromatography with a PD-10 column (GE Healthcare). Purified human RNase H2 was stored at −80°C in 50 mM Tris-HCl buffer (pH7.5) containing 200 mM KCl and 50% (v/v) glycerol before use. The enzyme concentration was determined using the molar absorption coefficient at 280 nm of 83,030 M^-1^ cm^-1^.

### SDS-PAGE

SDS-PAGE was performed in a 12.5% polyacrylamide gel under reducing conditions. Proteins were reduced by treatment with 2.5% of 2-mercaptoethanol at 100°C for 10 min, and then applied onto the gel. A constant current of 40 mA was applied for 40 min. After electrophoresis, proteins were stained with Coomassie Brilliant Blue R-250.

### Measurement of RNase H2 activity

A DNA_14_-RNA_1_-DNA_3_/DNA_18_ hybrid (named R1/D18) and an RNA_18_/DNA_18_ hybrid (named R18/D18) [19] were used as a substrate. They were prepared by incubating 1.0 μl of 100 μM 3’-FITC labeled 5’-GATCTGAGCCTGGGaGCT-FITC-3’ (R1) or 3’-FITC labeled 18-mer RNA 5’-gaucugagccugggagcu-FITC-3’ (R18) and 1.2 μl of 100 μM 5’-Dabcyl labeled 18-mer DNA 5’-Dabcyl-AGCTCCCAGGCTCAGATC-3’ (D18) (Fasmac, Atsugi, Japan) in 50 mM Tris-HCl buffer (pH 8.0) containing 60 mM KCl at 25°C for 30 min. R18/D18. The activity was measured as described previously [20, 24]. Briefly, the reaction was started by adding 20 μl of RNase H2 solution (0.03–350 nM) to the 180 μl of 50 mM Tris-HCl buffer (pH 8.0), 5 mM MgCl_2_, 0–200 mM KCl, either of 5.6 nM R1/D18 or 5.6 nM R18/D18 in a 96-well plate at 25°C. The reaction was monitored by following the increase in fluorescence intensity at 515 nm with excitation at 490 nm with an EnSight (PerkinElmer, Waltham, MA) every 5 s for 5 min.

### Circular dichroism (CD) measurement

CD measurement was performed as described previously [23]. Briefly, a Jasco J-820 (Tokyo, Japan) spectropolarimeter equipped with a Peltier system of cell temperature control was used. The spectrometer conditions were: spectral range 200–250 nm; 100 mdeg sensitivity; 0.2 nm resolutions; 4 s response time; 20 nm min^−1^ scan rate; and 5 accumulations. The control baseline was obtained with solvent and all the components without RNase H2. CD spectra were recorded at 25°C using 2-mm cell. The concentration of RNase H2 was 1.0 μM in 5 mM Tris-HCl buffer (pH 8.3), 20 mM KCl, 5% glycerol. CD spectra were processed with a Jasco software, and finally expressed in mean-residue molar ellipticity units, [*θ*] (deg cm^2^ dmol^−1^).

For the analysis of thermal denaturation of RNase H2, the solution (500 μl) containing RNase H2 (1.0 μM) in 5 mM Tris-HCl buffer (pH 8.3), 20 mM KCl, 5% glycerol was incubated at 25°C for 5 min. After the incubation, the solution (400 μl) was transferred to a 2-mm cell, and mineral oil (50 μl) was added to avoid evaporation. Thermal denaturation was examined by monitoring the CD value at 222 nm, *θ*_222_, from 30 to 70°C at 1°C/min. Fraction unfolded (*F*_u_) was determined after normalizing *θ*_222_ of native and denatured RNase H2 between 0 and 1, according to Eq (1).

$$F_{u}=\left(A_{O}-A_{N}\right)/\left(A_{D}-A_{N}\right)$$ (1)

where *A*_O_ is the observed *θ*_222_ of RNase H2 at various temperatures, and *A*_N_ and *A*_D_ are *θ*_222_ of native and denatured enzymes, respectively. The melting temperature (*T*_m_) was defined as the one at which *F*_u_ is 0.5.

## Results

### Location of Val143 in the active site of human RNase H2

Val143 is located in the small loop (Val143-Gly144-Met145) connecting the β sheet (Val135–Thr142) that contains Asp141 of the DEDD motif and the α helix (Pro146–Ser156) that is closely located to Arg38 of the GRG motif (Fig 1). Val143 is conserved only in eukaryotic RNase H2 (Fig 2). We thus hypothesized that Val143 plays an important role and explored its role by saturation mutagenesis. S1 and S2 Figs show the modelled human RNase H2 complexed with an RNA/DNA hybrid, suggesting that Val143 is located near the sugar moiety of the deoxyribonucleotide at the 5’ side of the scissile phosphodiester bond of the substrate although they are crude modeling.

### Production of recombinant human RNase H2 variants

We tried to express and purify 19 Val143 variants from *E*. *coli*. However, purified preparations were not obtained for V143C and V143M (S3 Fig). This might be due to unfavorable structural damage caused by the substitution of sulfur-containing amino acids.

**Fig 3:**
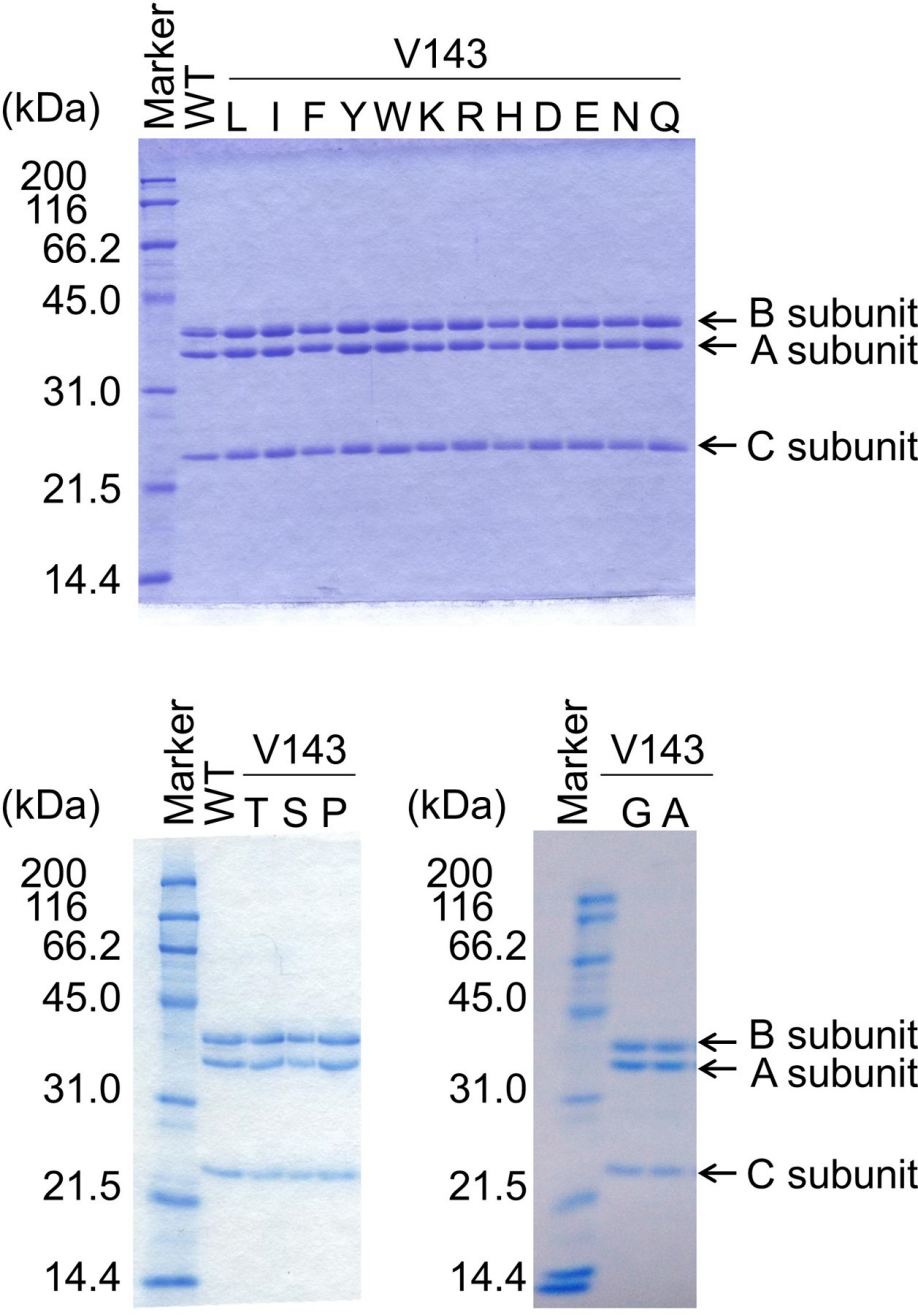
SDS-PAGE of Val143 variants under reducing conditions. Coomassie Brilliant Blue-stained 12.5% SDS-polyacrylamide gel showing marker proteins (Protein Markers for SDS-PAGE, Nacalai Tesque) and purified enzyme preparations of WT and 17 Val143 variants. The original gels are shown in S4 Fig.

Fig 3 shows the SDS-PAGE pattern of the preparations of the wild-type human RNase H2 (WT) and other 17 variants under reducing conditions. WT and all variants yielded three bands with molecular masses of 36, 34, and 24 kDa, corresponding to the B (theoretically 35.6 kDa), A (34.2 kDa), and C (18.6 kDa) subunits, respectively. The discrepancy between the calculated size of subunit C and the observed size was previously reported although the reason is unknown [5].

### Effects of mutation of Val143 on the RNase H2 activity

We analyzed the effects of mutation of Val143 on RNase H2 activity by the fluorescence-based assay with R1/D18 and R18/D18 as substrates. These substrates are designed to emit fluorescence when cleaved by RNase H2. The KCl concentration was set as 60 mM based on our previous report [20]. The initial reaction rate was obtained by following the increase in fluorescence intensity of the reaction solution. S5 Fig. shows the dependences on the enzyme concentration of the initial reaction rate. In both substrates, the initial reaction rates of WT and all 17 variants increased linearly with increasing enzyme concentration. However, the slope differed depending on variants and substrates.

Table 1 shows summary of the results shown in S5 Fig. When the activity of WT was set as 100%, the relative activities for R1/D18 of the 17 variants were in the range of 0.05–130%, and those for R18/D18 were in the range of 0.02–42%. In the hydrolysis of R1/D18, V143I exhibited the highest activity followed by WT. In the hydrolysis of R18/D18, WT exhibited the highest activity followed by V143I. When the ratio of the relative activity for R1/D18 to the relative activity for R18/D18 of WT was set as 1, the ratios of the 17 variants were in the range of 0.2–5.7, indicating that the substrate specificity varied depending on variants.

**Table 1:** Activities of Val143 variants.

	Initial reaction rate/Enzyme concentration×1,000 (s^-1^)				
Val143 variants	R1/D18 (A)		R18/D18 (B)		A/B
WT	307 ± 12	1.0^a^	235 ± 6	1.0^a^	1.31 (1.0)^b^
V143G	0.237 ± 0.016	0.00077	0.0721 ± 0.0039	0.00031	3.29 (2.7)
V143A	63.1 ± 2.9	0.21	19.4 ± 0.7	0.083	3.25 (2.6)
V143L	129 ± 1	0.42	35.0 ± 0.7	0.15	3.69 (2.8)
V143I	399 ± 1	1.3	98.7 ± 3.0	0.42	4.04 (3.1)
V143F	43.6 ± 3.0	0.14	14.8 ± 0.2	0.063	2.95 (2.3)
V143Y	97.9 ± 3.3	0.32	13.3 ± 0.5	0.057	7.36 (5.6)
V143W	79.3 ± 2.8	0.26	11.2 ± 0.4	0.048	7.08 (5.5)
V143K	0.517 ± 0.009	0.0017	0.0673 ± 0.0049	0.00029	7.68 (5.7)
V143R	0.153 ± 0.009	0.00050	0.0562 ± 0.0009	0.00024	2.72 (2.5)
V143H	4.39 ± 0.02	0.014	1.42 ± 0.06	0.0060	3.09 (2.3)
V143D	0.401 ± 0.024	0.0013	0.286 ± 0.006	0.0012	1.40 (1.1)
V143E	0.750 ± 0.020	0.0024	0.135 ± 0.005	0.00057	5.56 (4.0)
V143N	27.4 ± 1.1	0.089	5.56 ± 0.14	0.024	4.93 (3.9)
V143Q	2.49 ± 0.03	0.0081	7.90 ± 0.17	0.034	0.315 (0.2)
V143T	5.55 ± 0.31	0.018	3.87 ± 0.07	0.016	1.43 (1.1)
V143S	0.950 ± 0.130	0.0031	0.187 ± 0.001	0.00080	5.08 (3.9)
V143P	0.524 ± 0.068	0.0017	0.167 ± 0.001	0.00071	3.14 (2.4)

The reaction was carried out in 50 mM Tris-HCl buffer (pH 8.0), 5 mM MgCl_2_, 60 mM KCl, 5.6 nM R1/D18 or 5.6 nM R18/D18 at 25°C.

^a^Numbers indicate values relative to WT.

^b^Numbers in parentheses indicate values relative to WT.

**Fig 4:**
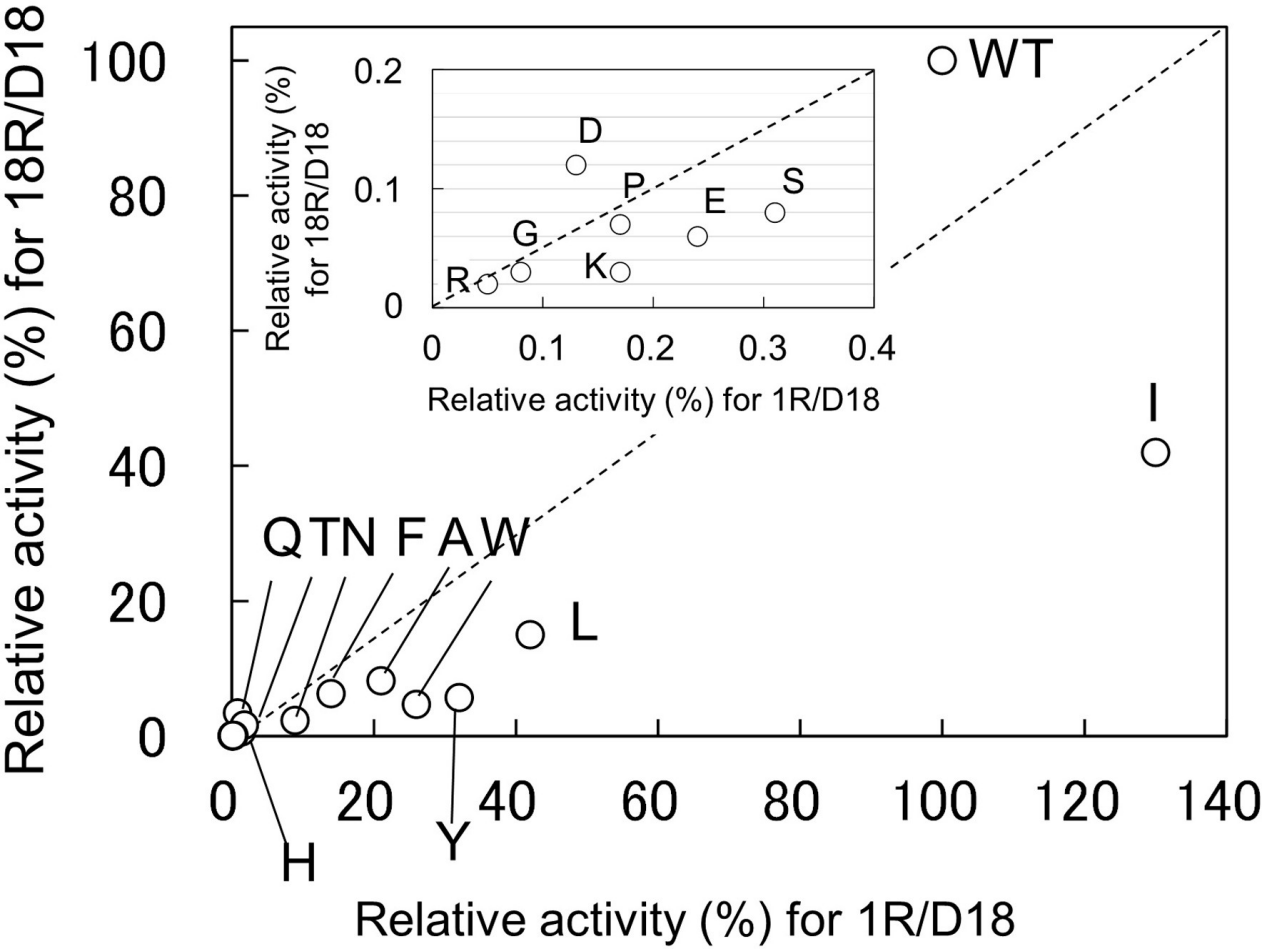
Comparison of the R1/D18-hydrolytic activity with the R18/D18-hydrolytic activity of Val143 variants. The reaction was carried out in 50 mM Tris-HCl buffer (pH 8.0), 5 mM MgCl_2_, 60 mM KCl, 5.6 nM R1/D18 or 5.6 nM R18/D18 at 25°C. Relative activities, which were activities relative to WT, in the hydrolysis of R1/D18 and R18/D18 were plotted.

For the grouping of variants, the hydrolytic activities of WT and the 17 variants for R1/D18 and R18/D18 were compared (Fig 4). All variants can be divided into three groups according to the residue into which Val143 was substituted: (i) Variants with charged residues at position 143 (V143D, V143E, V143K, V143H, and V143R). Their relative activities for R1/D18 and R18/D18 were markedly reduced (less than 2% of that of WT). (ii) Variants with bulky hydrophobic (V143F, V143I, V143L, and V143W) or bulky polar (V143Y) residues at position 143. Their relative activities for R1/D18 and R18/D18 were moderate (15–130% of that of WT). (iii) Variants with non-bulky hydrophobic (V143A and V143G) or non-bulky polar (V143N, V143P, V143Q, V143S, and V143T) residues at position 143. Their relative activities for R1/D18 and R18/D18 were markedly reduced (less than 2% of that of WT). In order to examine the effects of the amino acid residue at position 143 on the activity and structure of human RNase H2, we selected V143D and V143K from group (i), V143I and V143Y from group (ii), and V143G and V143N from group (iii) for subsequent analyses.

### Effects of the mutation at position 143 on the salt-dependence of RNase H2 activity

We previously analyzed the salt-dependence of the R1/D18- and R18/D18-hydrolyzing activities of WT [20]: NaCl, KCl, RbCl, and NaBr increased the activity to 170–390%, while LiCl, LiBr, and CsCl inhibited it, suggesting that species of cation, but not anion, is responsible for activity.

**Fig 5:**
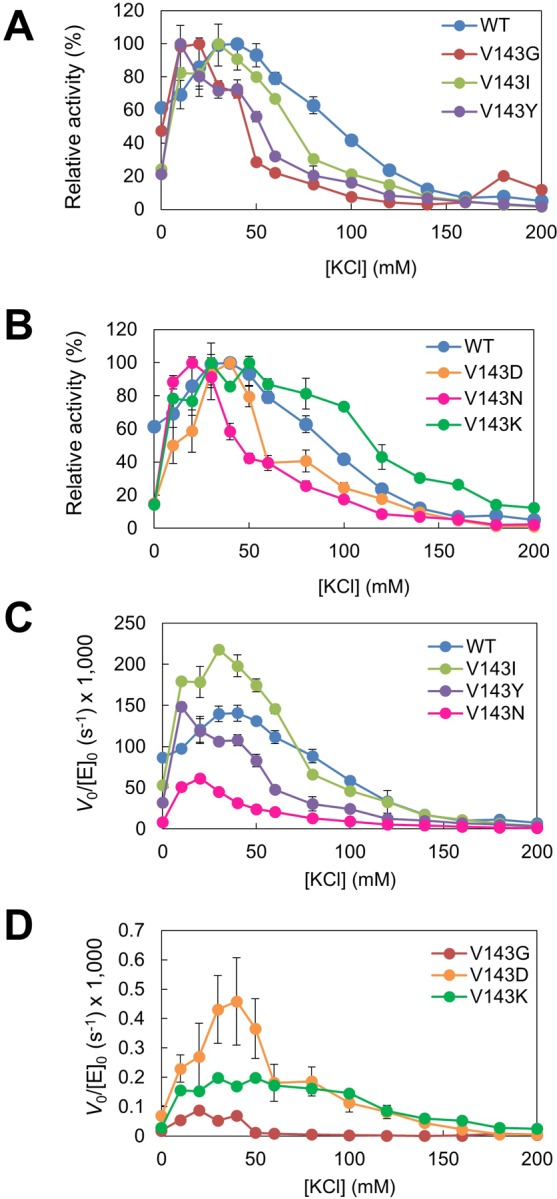
Dependence of activity of Val143 variants on KCl concentration for R1/D18. The reaction was carried out with 5.6 nM R1/D18 in 50 mM Tris-HCl buffer (pH 8.0), 5 mM MgCl_2_, 0–200 mM KCl at 25°C. Relative activity (A, B), which was defined as the ratio of the activity to the highest activity, and the initial reaction rates per enzyme concentration (C, D) against KCl concentration are shown. Error bars indicate SD values of triplicate determination. The original data are in S2 Table.

**Fig 6:**
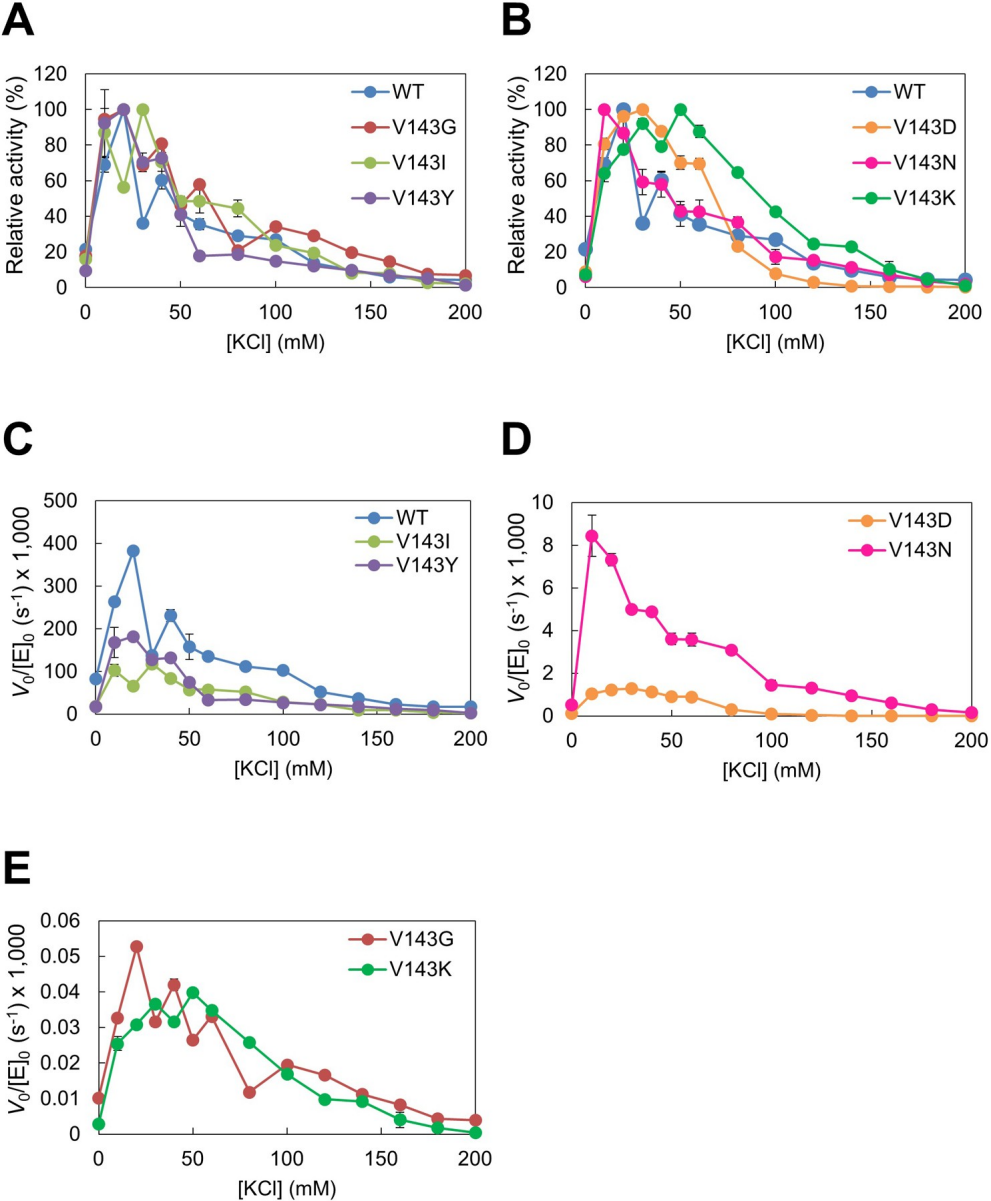
Dependence of activity of Val143 variants on KCl concentration for R18/D18. The reaction was carried out with 5.6 nM R18/D18 in 50 mM Tris-HCl buffer (pH 8.0), 5 mM MgCl_2_, 0–200 mM KCl at 25°C. Relative activity (A, B), which was defined as the ratio of the activity to the highest activity, and the initial reaction rates per enzyme concentration (C–E) against KCl concentration are shown. Error bars indicate SD values of triplicate determination. The original data are in S3 Table.

We analyzed the effects of KCl on the hydrolysis of R1/D18 (Fig 5) and R18/D18 (Fig 6). Relative activity was defined as the ratio of the activity to the highest activity. The relative activities of WT and variants were high at 20–50 mM KCl for R1/D18 and R18/D18, indicating that there was no difference in the KCl concentration at which the enzyme exhibited high activity between WT and variants.

### Effects of the mutation at position 143 on the stability of RNase H2

**Fig 7:**
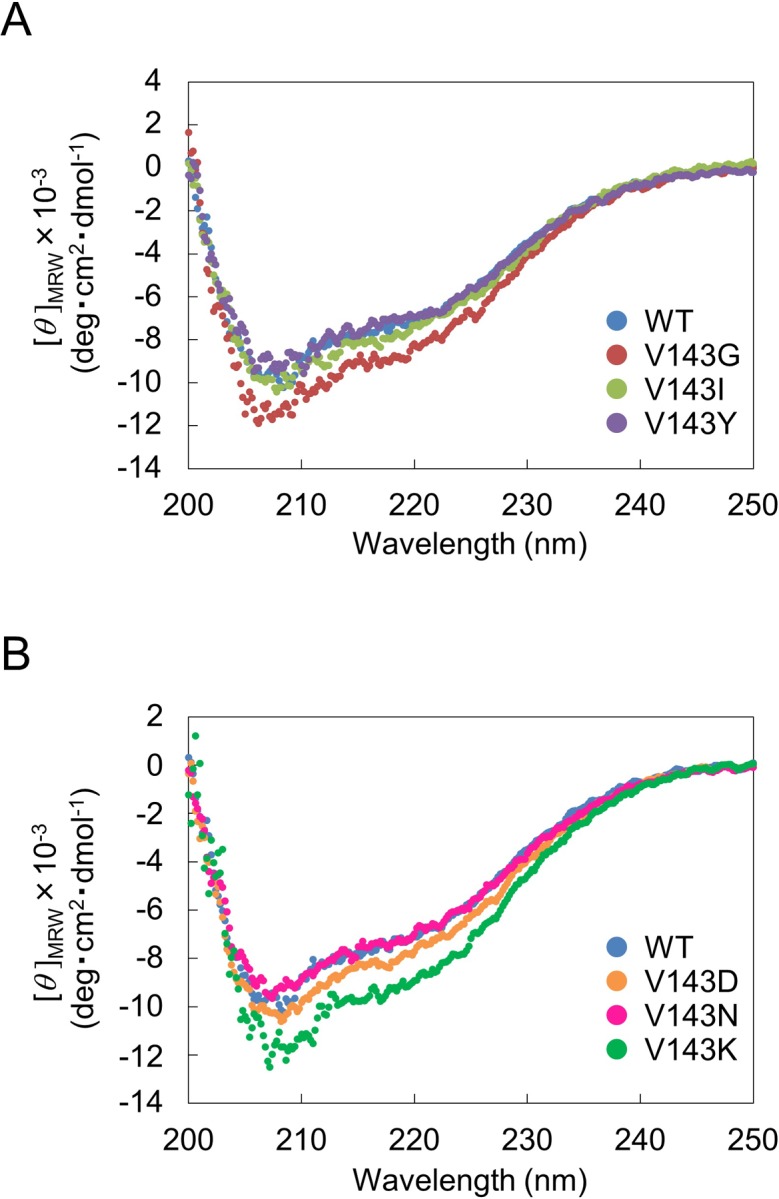
CD spectra of Val143 variants. The spectra at 200–250 nm were measured in 5 mM Tris-HCl buffer (pH 8.3), 20 mM KCl, 5% glycerol at 25°C with protein concentrations of 1.0 μM. One of the representative data of duplicate determination is shown. The original data are in S4 Table.

**Fig 8:**
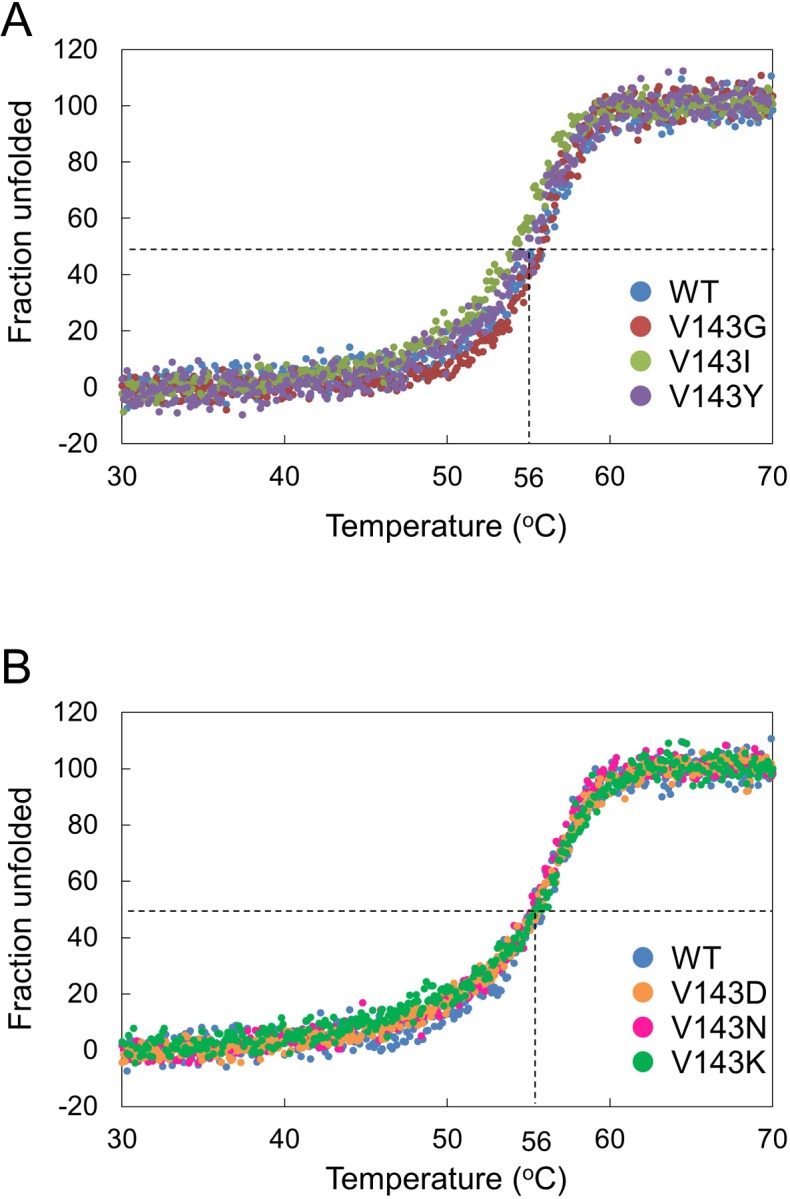
Thermal denaturation of Val143 variants. *θ*_222_ of WT and variants were monitored from 30 to 70°C at 1°C/min. The original data are in S5 Table.

First, we analyzed the secondary structure of WT and six variants by CD spectroscopy (Fig 7). All exhibited negative ellipticities at 200–250 nm. No appreciable changes were observed in each spectrum between WT and variants. Next, we analyzed the stability of WT and six variants by monitoring *θ*_222_ in the range of 30–70°C (Fig 8). The melting temperature (*T*_m_) was defined as where the Fraction unfolded (*F*_u_) is 0.5. The *T*_m_ values of WT and all variants were approximately 56°C, and the differences in *T*_m_ values were in the range of 1°C. These results suggested that except for the mutation to Cys or Met, the mutation at position 143 neither caused drastic structural changes nor made drastic reduction in stability.

## Discussion

In this study, we explored a role of Val143 of human RNase H2 in the activity and stability. Val143 is located in the small loop (Val143-Gly144-Met145) connecting the β sheet (Val135–Thr142) and the α helix (Pro146–Ser157) (PDB accession code 3PUF [7]) (Fig 1). Val143 of human RNase H2 is located at the active sire and is conserved in eukaryotic RNase H2. However, this valine residue has not been identified as an important residue for catalytic mechanism of human RNase H2.

The catalytic mechanism of human RNase H2 has been described as follows. Two metal ion mechanism of DNA polymerase [25], in which two divalent metal ions are present in the active site and are coordinated by the carboxylate of the conserved aspartate residues and the α-phosphate oxygen of the incoming dNTP, can be applied to RNase H [18], considering that the α-phosphate oxygen of the incoming dNTP corresponds to the oxygen of the scissile phosphodiester bond. In the crystal structure of the complex of *Thermotoga maritima* RNase HII and a DNA_5_-RNA_1_-DNA_6_/DNA_12_ hybrid, two Mg^2+^ ions in the active site are coordinated by Asp18, Glu19, Asp107, and Asp124 of the DEDD motif; Lys47 of the DSK motif interacts with the non-bridging oxygen of the scissile phosphodiester bond; and Arg22 of the GRG motif and Tyr163 interact with the oxygen of 2’-OH of the sugar moiety of the ribonucleotide at the 3’ side of the scissile phosphodiester bond [21]. We estimated that by thermodynamic analysis, pH and temperature dependence of human RNase H2 activity, ionizable groups responsible for acidic p*K*_e_ may be two of the three Asp34, Glu35 and Asp141 of DEDD motif, and that for alkaline p*K*_e_ may be Lys69 of DSK motif [20]. Thus, the following catalytic mechanism can be proposed for human, and probably other eukaryotic, RNase H2. In the absence of substrate, the enzyme Asp34, Glu35, and Asp141 of DEDD motif must be in their deprotonated state to coordinate two Mg^2+^ ions, and Lys69 must be in their unionized state for catalysis. Michaelis complex is formed, in which Arg38 of the GRG motif, Tyr210 that is a counterpart of Tyr163 in *T*. *maritima* RNase HII, and Lys69 of the DSK motif are involved in the binding with an RNA/DNA hybrid. Mg^2+^ ion polarizes the phosphodiester bond by coordinating to the non-bridging oxygen of the scissile phosphodiester bond. The complex in the transition state is formed when the ionized Mg^2+^-bound water attacks the phosphorus of the scissile bond. The phosphodiester bond is incised when the proton that binds to the non-bridging oxygen of the scissile phosphodiester bond is transferred to the binding oxygen of the scissile phosphodiester bond. In this mechanism, Val143 is not revealed as an important residue.

All Val143 variants except for V143C and V143M retained activity (Table 1). Variants in which Val143 is replaced with charged residues (V143D, V143E, V143K, V143H, and V143R) (group A) and those in which Val143 is replaced with non-bulky hydrophobic or non-bulky polar (V143A, V143G, V143N, V143P, V143Q, V143S, and V143T) (group B) exhibited markedly reduced activity (less than 2% of that of WT) (Table 1 and Fig 4). Considering that Val143 is located in the small loop (Val143-Gly144-Met145) connecting the β sheet (Val135–Thr142) that contains Asp141 of the DEDD motif and the α helix (Pro146–Ser156) (Fig 1), the decrease in activity in group A might be because the mutation altered the geometry of Arg38, resulting in the decrease in the binding ability of the enzyme to the substrate, and the decrease in activity in group B might be because the mutation increased the flexibility of this loop and altered the geometry of Asp141, resulting in the decrease in catalytic activity. To address this issue, steady-state kinetic analysis for separately obtaining *k*_cat_ and *K*_m_ values is necessary.

Salt-dependence of activity revealed that the magnitude of activation by KCl of all variants examined were 1.3–5 fold more than WT. This could be due to the mutation at position 143 causing unfavorable electrostatic repulsion with the substrate, possibly through changing the geometry of Arg38 and/or Asp141. Neutral salts may alleviate this repulsion to some extent. Taken together, these results indicated that Val143 is not critical but plays a role in determining catalytic activity.

Variants in which Val143 is replaced with bulky hydrophobic (V143F, V143I, V143L, and V143W) or bulky polar (V143Y) residues at position 143 exhibited moderate activity (14–130%) for the hydrolysis of R1/D18, suggesting that the substitution did not affect the geometry of Asp141. Interestingly, the substitution altered the ratios of R1/D18-hydrolyzing activity to R18/D18-hydrolyzing activity. When the ratio of WT was set as 1, the ratios of V143Y and V143W were 5.6 and 5.5, respectively, and that of V143Q was 0.2, suggesting the substitution affected the specificity. The alteration observed in V143Y and V143W might be because the bulky side chains caused steric hindrance with the 2’-OH of the sugar moiety of the ribonucleotide at the 5’ side of the scissile phosphodiester bond.

In this study, all Val143 variants examined exhibited similar secondary structure (Fig 7) and thermostability (Fig 8) to WT, indicating that Val143 does not play a role in stability. Val143 is present in the A subunit and is not involved in the association with the B or C subunit thereby limiting its influence on structure and stability. In the severe neuroinflammatory disorder Aicardi-Goutières syndrome, more than 50% of total AGS patients have biallelic mutations in one of the three genes encoding RNase H2: 5% for *RNASEH2A*, 36% for *RNASEH2B*, and 12% for *RNASEH2C* [26]. The mutation of Val143 has not been observed in AGS. It was reported that a number of recombinant RNase H2 variants bearing AGS-causing mutations exhibited reduced stability and/or hetrotrimer forming ability [5–7, 17, 23, 27, 28].

According to the genomic analyses of esophageal squamous cell carcinoma (ESCC) cells isolated from 104 patients, the mutation of G427A was found in *RNASEH2A* in chromosome 19, which corresponds to the mutation of V143I in the A subunit of human RNase H2 [29]. To elucidate the relationships of the mutations of RNase H2 genes and diseases, further study is required.

In conclusion, we performed saturation mutagenesis analysis of Val143 of the A subunit in human RNase H2. The results revealed that Val143 is not critical for catalytic activity but fine-tunes the activity and specificity of human RNase H2.

## Supporting information

S1 Fig Modelled structure of the a subunit of human RNase H2 complexed DNA_5_-RNA_1_-DNA_6_/DNA_12_. (A) Overall structure. The A subunit of human RNase H2 is shown as ribbon in green, and Pro40, Val143, and Tyr210 are shown as sticks in cyan, orange, and magenta, respectively. Deoxyribonucleotide and ribonucleotide in the scissile strand of the hybrid are shown as sticks in blue and red, respectively. Deoxyribonucleotide in another strand of the hybrid is not shown. (B) Close-up view of the active site. The arrow indicates the site of cleavage.
(PDF)

S2 Fig Modelled structure of the a subunit of human RNase H2 complex with RNA_19_/DNA_19_. (A) Overall structure. The colors of the structure correspond to S1 Fig. (B) Close-up view of the active site. The arrow indicates the site of cleavage.
(PDF)

S3 Fig SDS-PAGE under reducing conditions. Coomassie Brilliant Blue-stained 12.5% SDS-polyacrylamide gels are shown. Active fractions of each purification stage for V143C and V143M were applied. Lanes: marker proteins (lane 1), soluble fractions of the total extracts (lane 2), active fractions of heparin affinity column chromatography (lane 3), active fractions of Ni^2+^ affinity chromatography (lane 4), and active fractions of gel filtration columns (lane 5).
(PDF)

S4 Fig SDS-PAGE under reducing conditions. Coomassie Brilliant Blue-stained 12.5% SDS-polyacrylamide gel showing marker proteins (Protein Markers for SDS-PAGE, Nacalai Tesque) and purified enzyme preparations of WT and 17 Val143 variants, which corresponds to the original gel of the one shown in Fig 3. Lanes X are not included in the final figure.
(PDF)

S5 Fig Comparison of the R1/D18-hydrolytic activity (open circle) with the R18/D18-hydrolytic activity (filled circle) of Val143 variants. (PDF)

S1 Table Primer sets for preparing the Val143 variants. (PDF)

S2 Table Dependence of activity of Val143 variants on KCl concentration for R1/D18. The original data of Fig 5 are shown.
(PDF)

S3 Table Dependence of activity of Val143 variants on KCl concentration for R18/D18. The original data of Fig 6 are shown.
(PDF)

S4 Table CD spectra of Val143 variants. The original data of Fig 7 are shown.
(PDF)

S5 Table Thermal denaturation of Val143 variants. The original data of Fig 8 are shown.
(PDF)
