# Supplementary material for: Val143 of human ribonuclease H2 is not critical for, but plays a role in determining catalytic activity and substrate specificity
Source: PLoS One. 2020 Feb 18;15(2):e0228774. doi: 10.1371/journal.pone.0228774 (PMC7028304; doi:10.1371/journal.pone.0228774)
Supplement: S1 Fig — (A) Overall structure. The A subunit of human RNase H2 is shown as ribbon in green, and Pro40, Val143, and Tyr210 are shown as sticks in cyan, orange, and magenta, respectively. Deoxyribonucleotide and ribonucleotide in the scissile strand of the hybrid are shown as sticks in blue and red, respectively. Deoxyribonucleotide in another strand of the hybrid is not shown. (B) Close-up view of the active site. The arrow indicates the site of cleavage. (PDF) [file pone.0228774.s001.pdf]

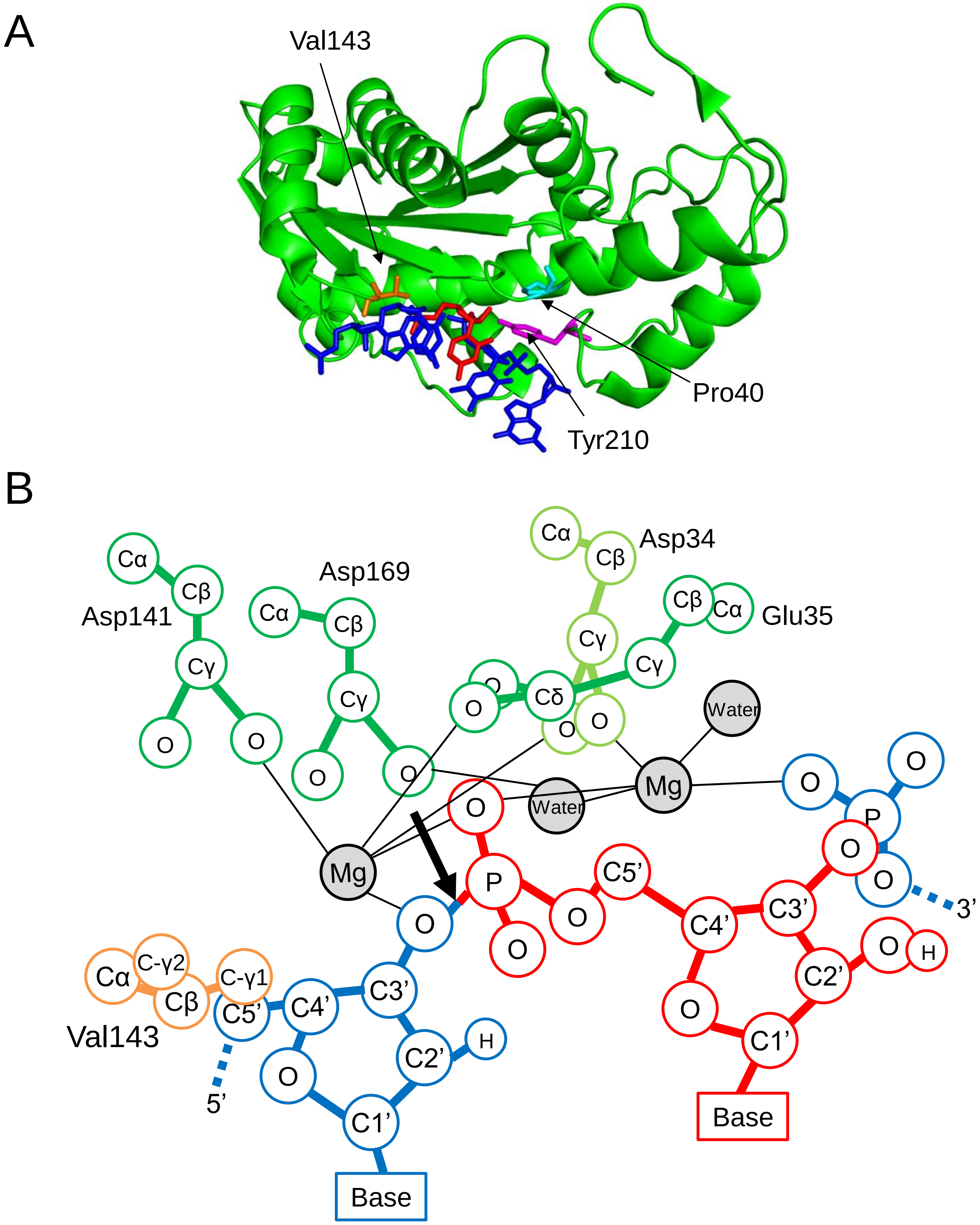

S1 Fig. Modelled structure of the A subunit of human RNase H2 complexed DNA_5-RNA_1-DNA_6/DNA_{12}. (A) Overall structure. The A subunit of human RNase H2 is shown as ribbon in green, and Pro40, Val143, and Tyr210 are shown as sticks in cyan, orange, and magenta, respectively. Deoxyribonucleotide and ribonucleotide in the scissile strand of the hybrid are shown as sticks in blue and red, respectively. Deoxyribonucleotide in another strand of the hybrid is not shown. (B) Close-up view of the active site. The arrow indicates the site of cleavage.

S1 Fig
