# Supplementary material for: Val143 of human ribonuclease H2 is not critical for, but plays a role in determining catalytic activity and substrate specificity
Source: PLoS One. 2020 Feb 18;15(2):e0228774. doi: 10.1371/journal.pone.0228774 (PMC7028304; doi:10.1371/journal.pone.0228774)
Supplement: S2 Fig — (A) Overall structure. The colors of the structure correspond to S1 Fig. (B) Close-up view of the active site. The arrow indicates the site of cleavage. (PDF) [file pone.0228774.s002.pdf]

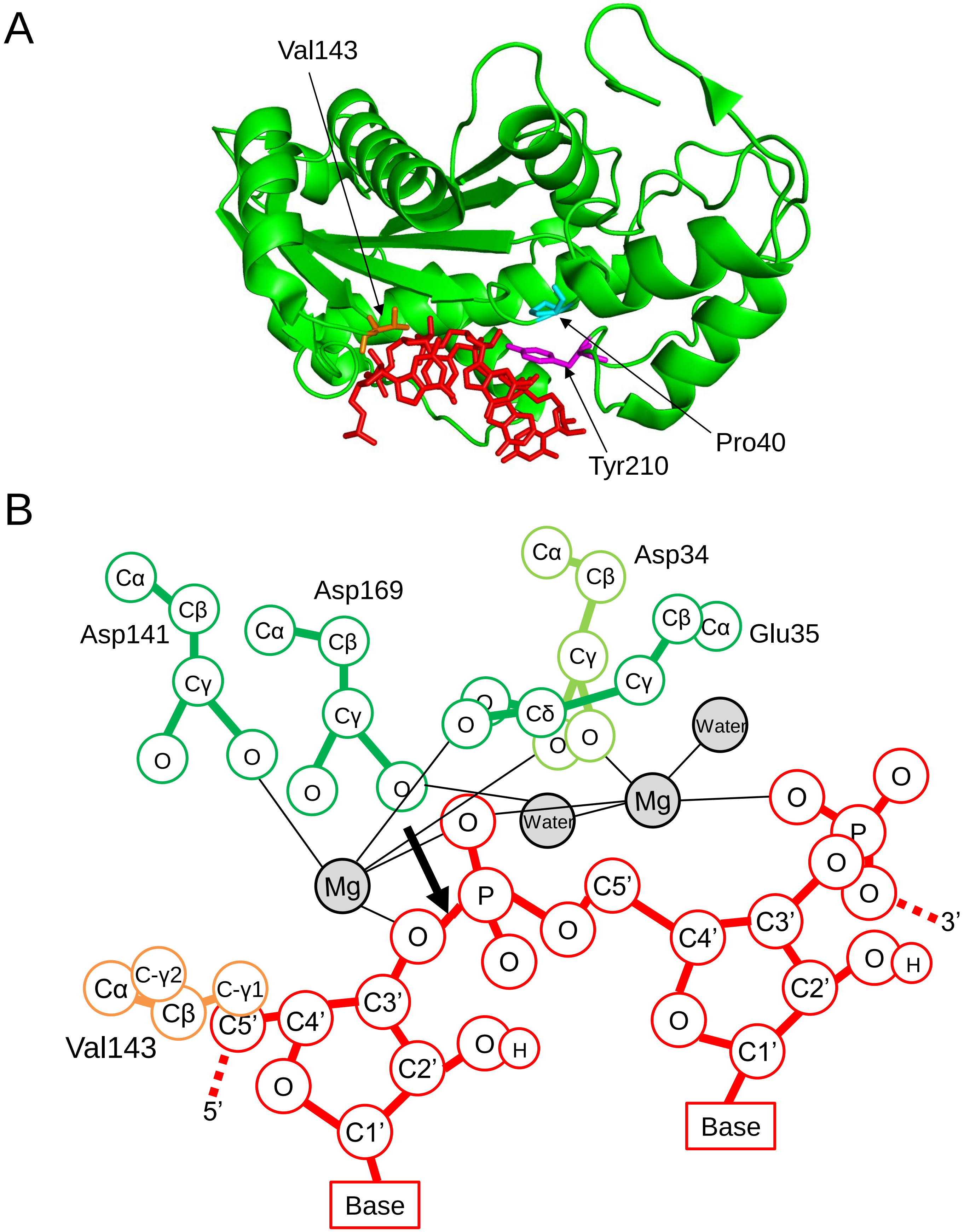

S2 Fig. Modelled structure of the A subunit of human RNase H2 complex with RNA_{19}/DNA_{19}. (A) Overall structure. The colors of the structure correspond to S1 Fig. (B) Close-up view of the active site. The arrow indicates the site of cleavage.

S2 Fig
