# Supplementary material for: Val143 of human ribonuclease H2 is not critical for, but plays a role in determining catalytic activity and substrate specificity
Source: PLoS One. 2020 Feb 18;15(2):e0228774. doi: 10.1371/journal.pone.0228774 (PMC7028304; doi:10.1371/journal.pone.0228774)
Supplement: S3 Fig — Coomassie Brilliant Blue-stained 12.5% SDS-polyacrylamide gels are shown. Active fractions of each purification stage for V143C and V143M were applied. Lanes: marker proteins (lane 1), soluble fractions of the total extracts (lane 2), active fractions of heparin affinity column chromatography (lane 3), active fractions of Ni2+ affinity chromatography (lane 4), and active fractions of gel filtration columns (lane 5). (PDF) [file pone.0228774.s003.pdf]

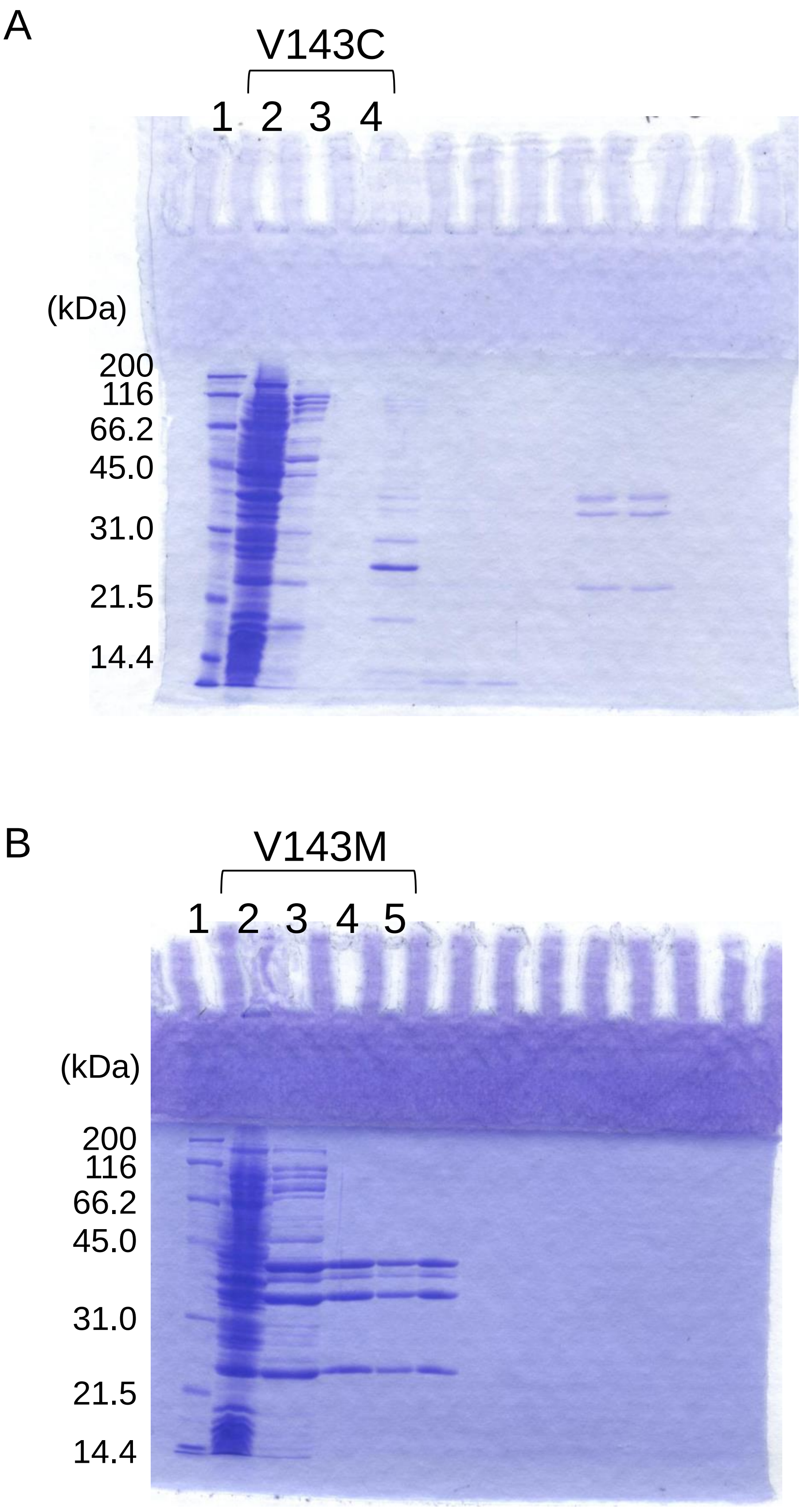

S3 Fig. SDS-PAGE under reducing conditions. Coomassie Brilliant Blue-stained 12.5% SDS-polyacrlamide gels are shown. Active fractions of each purification stage for V143C and V143M were applied. Lanes: marker proteins (lane 1), soluble fractions of the total extracts (lane 2), active fractions of heparin affinity column chromatography (lane 3), active fractions of Ni^{2+} affinity chromatography (lane 4), and active fractions of gel filtration columns (lane 5).

S3 Fig
