# Supplementary material for: Val143 of human ribonuclease H2 is not critical for, but plays a role in determining catalytic activity and substrate specificity
Source: PLoS One. 2020 Feb 18;15(2):e0228774. doi: 10.1371/journal.pone.0228774 (PMC7028304; doi:10.1371/journal.pone.0228774)
Supplement: S4 Fig — Coomassie Brilliant Blue-stained 12.5% SDS-polyacrylamide gel showing marker proteins (Protein Markers for SDS-PAGE, Nacalai Tesque) and purified enzyme preparations of WT and 17 Val143 variants, which corresponds to the original gel of the one shown in Fig 3. Lanes X are not included in the final figure. (PDF) [file pone.0228774.s004.pdf]

A

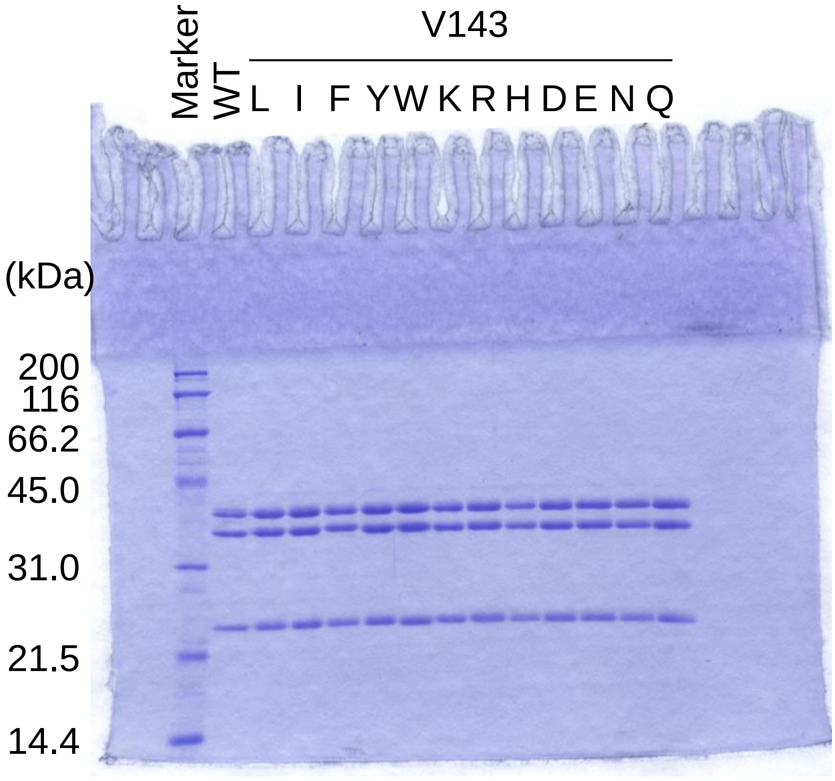
Marker
WT
V143
L I F Y W K R H D E N Q
(kDa)
200
116
66.2
45.0
31.0
21.5
14.4

B

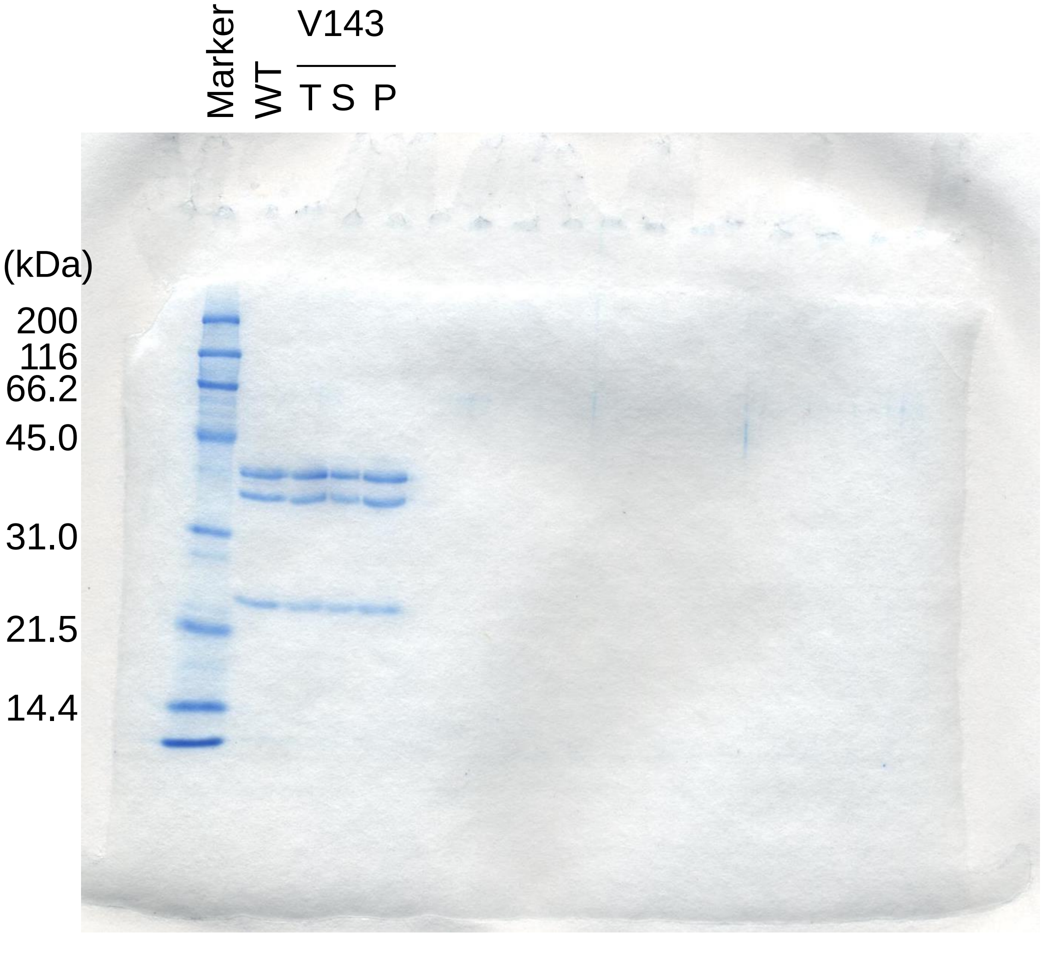
Marker
WT
V143
T S P
(kDa)
200
116
66.2
45.0
31.0
21.5
14.4

S4 Fig

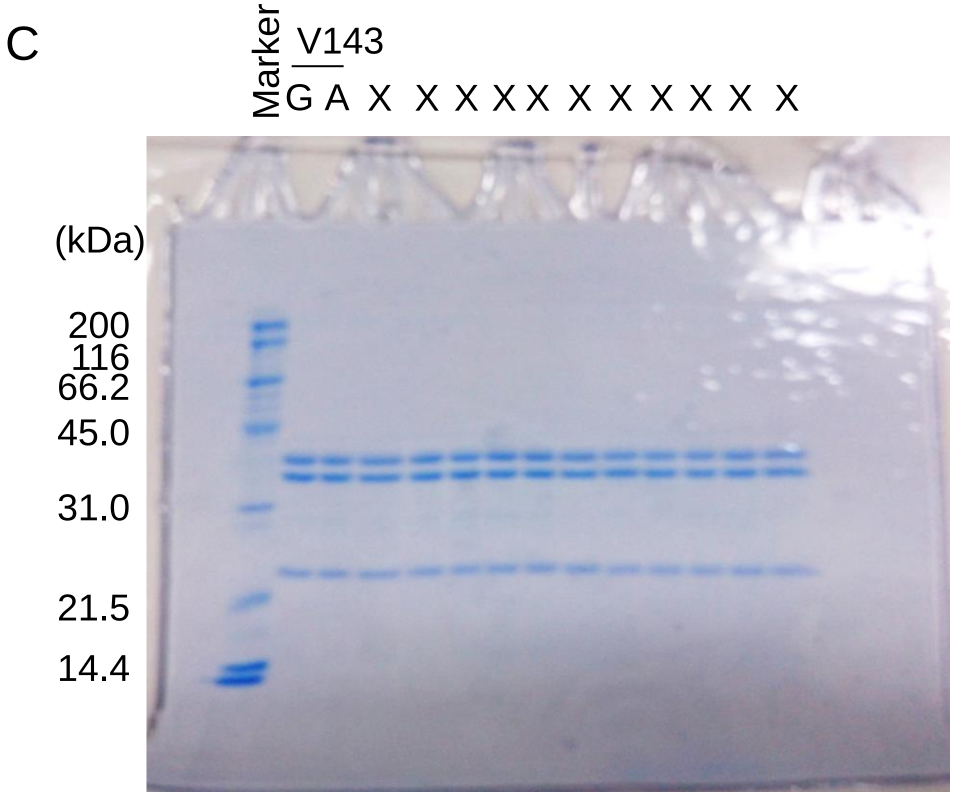

S4 Fig. SDS-PAGE under reducing conditions. Coomassie Brilliant Blue-stained 12.5% SDS-polyacrylamide gel showing marker proteins (Protein Markers for SDS-PAGE, Nacalai Tesque) and purified enzyme preparations of WT and 17 Val143 variants, which corresponds to the original gel of the one shown in Fig 3. Lanes X are not included in the final figure.
