# Supplementary material for: Val143 of human ribonuclease H2 is not critical for, but plays a role in determining catalytic activity and substrate specificity
Source: PLoS One. 2020 Feb 18;15(2):e0228774. doi: 10.1371/journal.pone.0228774 (PMC7028304; doi:10.1371/journal.pone.0228774)
Supplement: S5 Fig — (PDF) [file pone.0228774.s005.pdf]

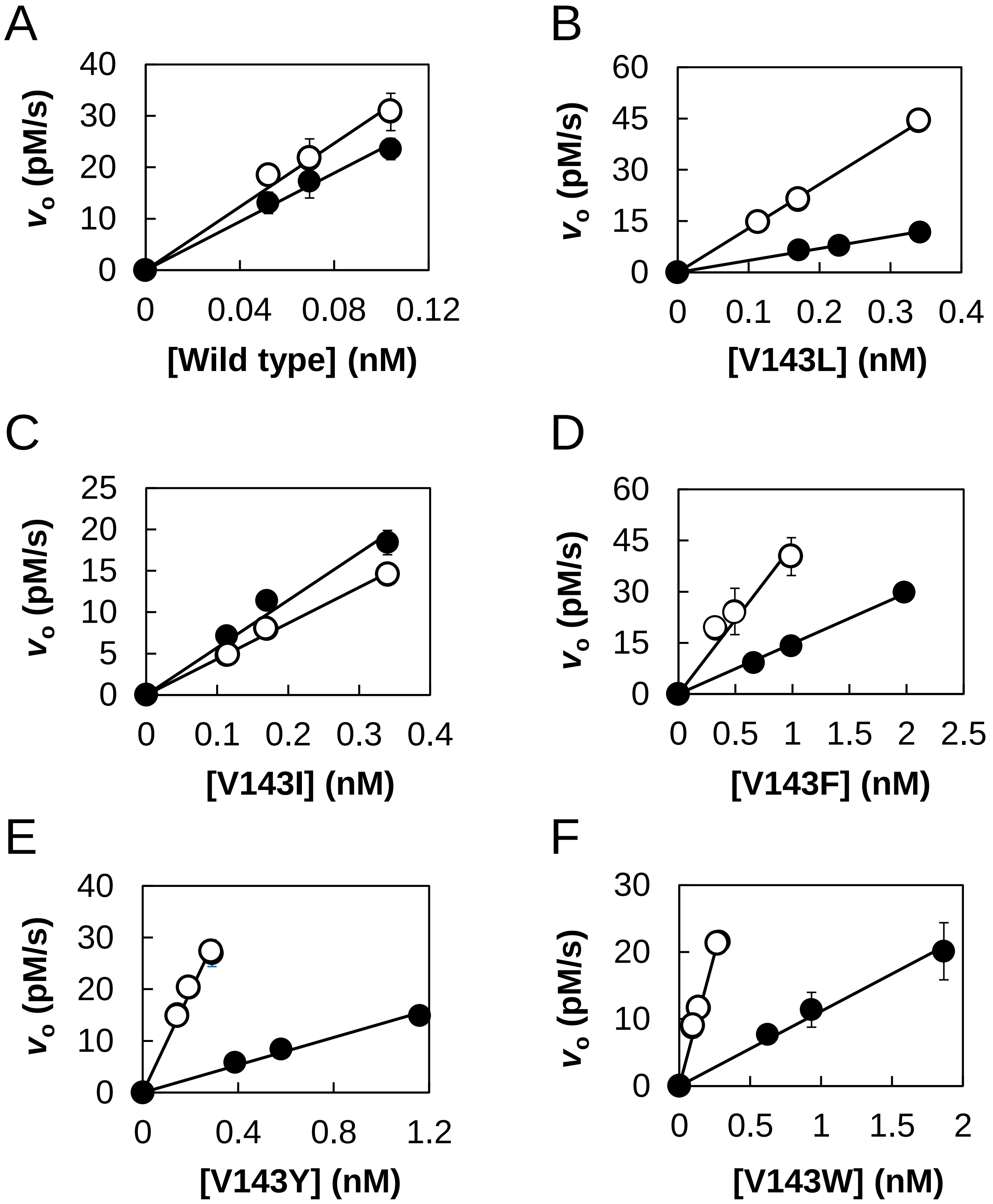

A
B
C
D
E
F
vo (pM/s)
[Wild type] (nM)
[V143L] (nM)
[V143I] (nM)
[V143F] (nM)
[V143Y] (nM)
[V143W] (nM)

S5 Fig

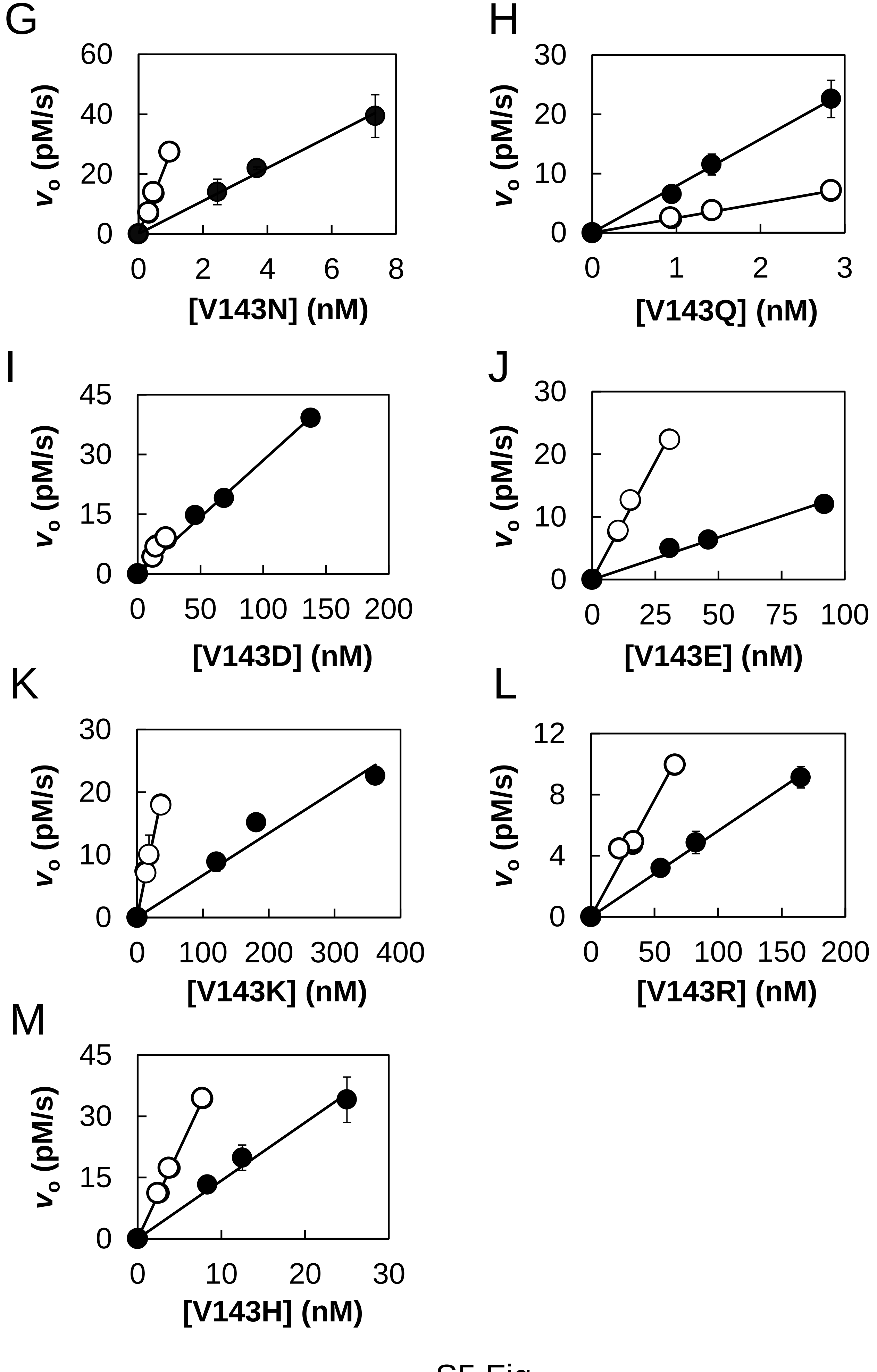
G
v_o (pM/s)
60
40
20
0
0
2
4
6
8
[V143N] (nM)
H
v_o (pM/s)
30
20
10
0
0
1
2
3
[V143Q] (nM)
I
v_o (pM/s)
45
30
15
0
0
50
100
150
200
[V143D] (nM)
J
v_o (pM/s)
30
20
10
0
0
25
50
75
100
[V143E] (nM)
K
v_o (pM/s)
30
20
10
0
0
100
200
300
400
[V143K] (nM)
L
v_o (pM/s)
12
8
4
0
0
50
100
150
200
[V143R] (nM)
M
v_o (pM/s)
45
30
15
0
0
10
20
30
[V143H] (nM)

S5 Fig

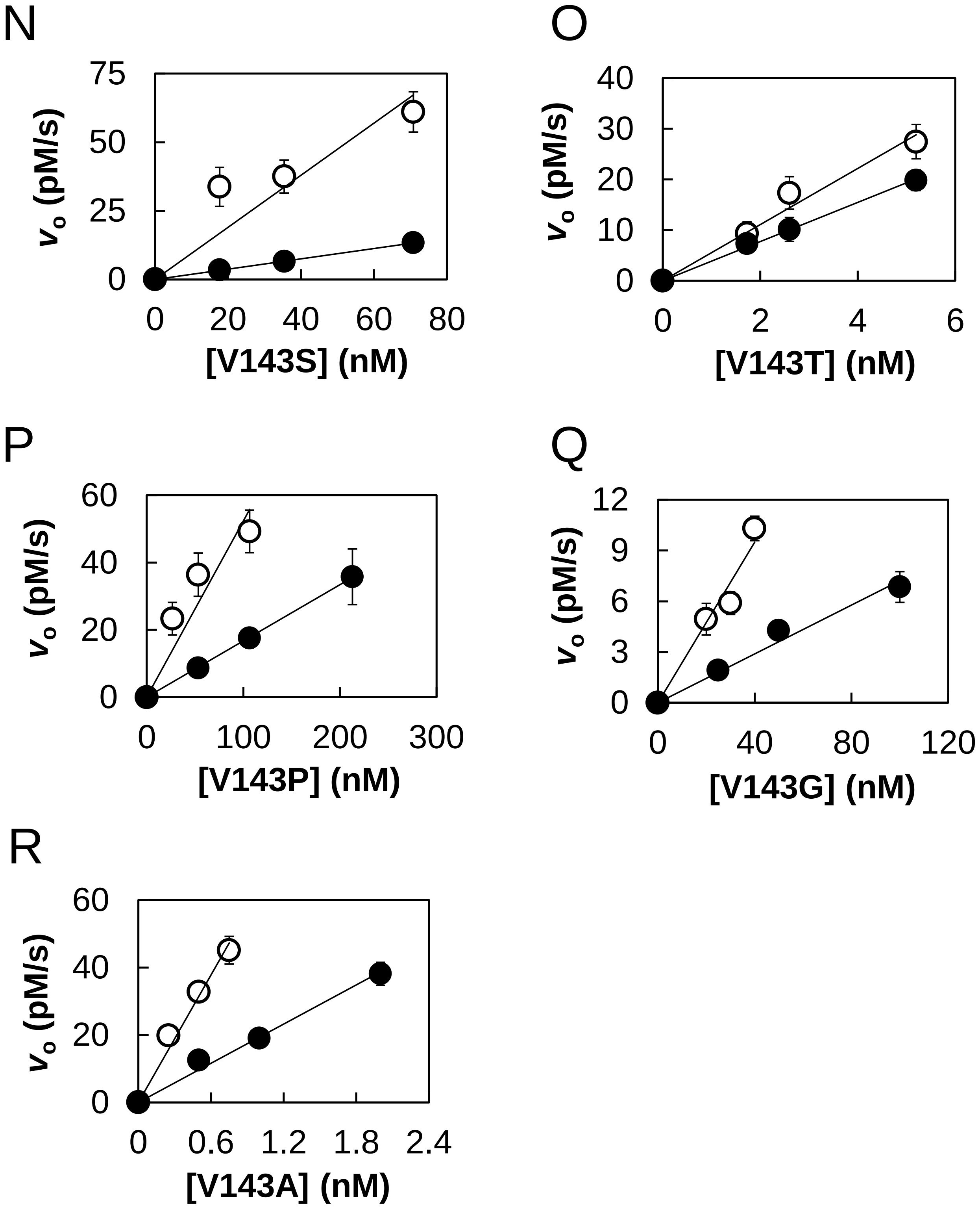

S5 Fig. Comparison of the R1/D18-hydrolytic activity (open circle) with the R18/D18-hydrolytic activity (filled circle) of Val143 variants.

S5 Fig
