# Supplementary material for: Val143 of human ribonuclease H2 is not critical for, but plays a role in determining catalytic activity and substrate specificity
Source: PLoS One. 2020 Feb 18;15(2):e0228774. doi: 10.1371/journal.pone.0228774 (PMC7028304; doi:10.1371/journal.pone.0228774)
Supplement: S1 Table — (PDF) [file pone.0228774.s006.pdf]

S1 Table. Primer sets for preparing the Val143 variants.

V143L_F	5’-CAGGTATTCGTGGACACCTTAGGGATGCCAGAGAC-3’
V143L_R	5’-GTCTCTGGCATCCCTAAGGTGTCCACGAATACCTG-3’
V143I_F	5’-CAGGTATTCGTGGACACCATTGGGATGCCAGAGAC-3’
V143I_R	5’-GTCTCTGGCATCCCAATGGTGTCCACGAATACCTG-3’
V143F_F	5’-CAGGTATTCGTGGACACCTTTGGGATGCCAGAGAC-3’
V143F_R	5’-GTCTCTGGCATCCCAAAGGTGTCCACGAATACCTG-3’
V143Y_F	5’-CAGGTATTCGTGGACACCTATGGGATGCCAGAGAC-3’
V143Y_R	5’-GTCTCTGGCATCCCATAGGTGTCCACGAATACCTG-3’
V143W_F	5’-CAGGTATTCGTGGACACCTGGGGGATGCCAGAGAC-3’
V143W_R	5’-GTCTCTGGCATCCCCCAGGTGTCCACGAATACCTG-3’
V143N_F	5’-CAGGTATTCGTGGACACCAACGGGATGCCAGAGAC-3’
V143N_R	5’-GTCTCTGGCATCCCGTTGGTGTCCACGAATACCTG-3’
V143Q_F	5’-CAGGTATTCGTGGACACCCAGGGGATGCCAGAGAC-3’
V143Q_R	5’-GTCTCTGGCATCCCCTGGGTGTCCACGAATACCTG-3’
V143D_F	5’-CAGGTATTCGTGGACACCGATGGGATGCCAGAGAC-3’
V143D_R	5’-GTCTCTGGCATCCCATCGGTGTCCACGAATACCTG-3’
V143E_F	5’-CAGGTATTCGTGGACACCGAGGGGATGCCAGAGAC-3’
V143E_R	5’-GTCTCTGGCATCCCCTCGGTGTCCACGAATACCTG-3’
V143K_F	5’-CAGGTATTCGTGGACACCAAAGGGATGCCAGAGAC-3’
V143K_R	5’-GTCTCTGGCATCCCTTTGGTGTCCACGAATACCTG-3’
V143R_F	5’-CAGGTATTCGTGGACACCCGTGGGATGCCAGAGAC-3’
V143R_R	5’-GTCTCTGGCATCCCACGGGTGTCCACGAATACCTG-3’
V143H_F	5’-CAGGTATTCGTGGACACCCATGGGATGCCAGAGAC-3’
V143H_R	5’-GTCTCTGGCATCCCATGGGTGTCCACGAATACCTG-3’

Nucleotide sequences for introducing mutations are underlined.
