# Supplementary material for: Val143 of human ribonuclease H2 is not critical for, but plays a role in determining catalytic activity and substrate specificity
Source: PLoS One. 2020 Feb 18;15(2):e0228774. doi: 10.1371/journal.pone.0228774 (PMC7028304; doi:10.1371/journal.pone.0228774)
Supplement: S2 Table — The original data of Fig 5 are shown. (PDF) [file pone.0228774.s007.pdf]

S2 Table. Dependence of activities of Val143 variants on KCl concentration for R1/D18.

[KCl]	$V_0/[E]_0$ (s^{-1}) × 1,000[a]						
(mM)	WT	V143I	V143G	V143D	V143K	V143Y	V143N
0	87±3.7	53±1.8	0.018 ±0.00057	0.069±0.034	0.028 ±0.00089	32±0.74	8.0±0.11
	(62±3.2)[b]	(24±0.53)	(47±2.7)	(15±1.4)	(14±0.50)	(21±1.4)	(15±1.1)
10	98±5.0	180±1.2	0.055 ±0.0039	0.23±0.046	0.16±0.0053	150±5.8	51±2.9
	(69±8.5)	(82±1.3)	(98±13)	(50±11)	(78±2.6)	(100)	(88±4.1)
20	120±16	180±19	0.088 ±0.0011	0.27±0.11	0.15±0.0040	120±15	61±5.0
	(86±18)	(82±7.4)	(100)	(59±13)	(77±2.1)	(80±7.4)	(100)
30	140±9.6	220±3.7	0.053 ±0.00055	0.43±0.12	0.20 ±0.00076	110±2.7	45±4.8
	(99±13)	(100)	(74±3.2)	(94±9.0)	(100)	(72±4.5)	(91±14)
40	140±9.7	200±13	0.070 ±0.00071	0.46±0.15	0.17±0.0047	110±6.8	31±0.53
	(100)	(91±6.6)	(71±1.4)	(100)	(86±2.2)	(73±5.5)	(58±5.0)
50	130±3.2	170±7.8	0.011 ±0.00034	0.37±0.10	0.20±0.0076	83±7.8	24±0.53
	(93±7.0)	(80±2.4)	(29±1.2)	(80±6.2)	(100±3.9)	(56±3.2)	(42±2.9)
60	110±7.7	150±5.7	0.0084 ±0.00048	0.18±0.063	0.17±0.0069	47±5.0	20±1.1
	(79±3.6)	(67±1.6)	(22±0.74)	(39±1.6)	(87±3.2)	(32±2.4)	(40±4.6)
80	88±8.2	66±3.7	0.0058 ±0.000024	0.19±0.049	0.16±0.018	30±8.8	13±0.82
	(63±5.1)	(30±1.9)	(15±0.49)	(41±6.6)	(81±9.3)	(20±5.7)	(26±3.0)
100	59±2.5	46±3.2	0.0029 ±0.00028	0.11±0.032	0.15±0.0049	24±2.0	8.8±0.66
	(42±3.1)	(21±0.76)	(7.5±0.71)	(25±2.9)	(74±2.2)	(16±1.1)	(18±2.1)
120	33±0.60	33±14	0.0017 ±0.00033	0.082±0.023	0.085±0.015	12±1.3	5.1±0.50
	(24±1.3)	(15±2.3)	(4.3±0.97)	(18±1.9)	(43±7.4)	(8.4±0.77)	(8.7±0.49)
140	17±0.42	17±0.53	0.0011 ±0.00033	0.045 ±0.0086	0.060 ±0.0026	9.9±0.93	3.9±0.19
	(12±1.1)	(7.6±0.23)	(2.9±0.90)	(9.8±3.4)	(30±1.3)	(6.6±0.83)	(7.0±0.61)

160	9.9±0.60<br>(7.1±0.80)	11±0.45<br>(5.0±0.070)	0.0017<br>±0.00047<br>(4.3±1.1)	0.023<br>±0.0057<br>(4.9±0.71)	0.052<br>±0.0016<br>(26±0.74)	6.9±0.32<br>(4.7±0.39)	2.4±0.44<br>(5.4±1.1)
180	11±0.43<br>(7.8±0.27)	6.1±0.32<br>(2.8±0.016)	0.0077<br>±0.00044<br>(20±1.7)	0.0061<br>±0.0053<br>(1.3±0.92)	0.028<br>±0.00050<br>(14±0.21)	5.0±0.090<br>(3.3±0.13)	1.2±0.083<br>(2.1±0.19)
200	7.1±0.20<br>(5.1±0.30)	3.7±0.54<br>(1.7±0.092)	0.0046<br>±0.00083<br>(12±2.5)	0.0055±0.00<br>32<br>(1.2±0.57)	0.025<br>±0.00064<br>(12±0.33)	2.9±0.37<br>(1.9±0.26)	0.80±0.33<br>(2.3±0.67)

[a]The reaction was carried out in 50 mM Tris-HCl buffer (pH 8.0), 5 mM $MgCl_2$, 5.6 nM R1/D18 at 25ºC.

[b]Numbers in parentheses indicate values relative to the highest activity in each variant.
