# Supplementary material for: Val143 of human ribonuclease H2 is not critical for, but plays a role in determining catalytic activity and substrate specificity
Source: PLoS One. 2020 Feb 18;15(2):e0228774. doi: 10.1371/journal.pone.0228774 (PMC7028304; doi:10.1371/journal.pone.0228774)
Supplement: S3 Table — The original data of Fig 6 are shown. (PDF) [file pone.0228774.s008.pdf]

S3 Table. Dependence of activities of Val143 variants on KCl concentration.

[KCl]	V_0/[E]$_0$ (s^{-1}) × 1,000[a]						
(mM)	WT	V143I	V143G	V143D	V143K	V143Y	V143N
0	83±5.0 (22±1.9)[b]	19±1.4 (16±1.0)	0.010 ±0.00094 (18±1.7)	0.12 ±0.0038 (9±0.32)	0.0029 ±0.00035 (7.2±0.62)	17±1.0 (9.5±0.38)	0.52 ±0.021 (6.2±0.86)
10	260±8.8 (69±4.1)	100±14 (87±13)	0.033 ±0.00081 (95±2.2)	1.0±0.048 (81±3.3)	0.025 ±0.0019 (64±4.6)	170±36 (92±19)	8.4±0.96 (100)
20	380±9.3 (100)	66±6.1 (56±1.7)	0.053 ±0.00061 (100)	1.2±0.016 (96±1.8)	0.031 ±0.00066 (78±2.4)	180±3.7 (100)	7.3±0.30 (87±11)
30	140±6.2 (36±2.5)	120±7.1 (100)	0.032 ±0.0013 (69±2.9)	1.3±0.019 (100)	0.037 ±0.00059 (92±2.9)	130±7.0 (70±5.3)	5.0±0.088 (59±7.2)
40	230±14 (60±4.9)	83±7.3 (71±8.5)	0.042 ±0.0016 (81±2.1)	1.1±0.030 (88±2.5)	0.032 ±0.00035 (79±1.6)	130±6.4 (73±3.2)	4.9±0.12 (58±7.0)
50	160±30 (41±7.0)	57±4.6 (48±2.8)	0.026 ±0.00058 (46±1.5)	0.90±0.043 (70±3.7)	0.040 ±0.00084 (100)	75±1.6 (41±1.3)	3.6±0.27 (43±3.6)
60	140±8.5 (36±3.1)	57±5.7 (48±6.5)	0.033 ±0.0011 (58±2.3)	0.89±0.42 (70±3.1)	0.035 ±0.0012 (88±3.5)	33±2.2 (18±1.5)	3.6±0.31 (42±6.6)
80	110±3.1 (29±1.5)	52±3.5 (45±4.8)	0.012 ±0.00029 (21±0.49)	0.30 ±0.012 (23±1.1)	0.026 ±0.0013 (65±2.4)	34±1.2 (19±0.84)	3.1±0.11 (37±3.0)
100	100±4.2 (27±0.80)	28±2.4 (24±1.7)	0.020 ±0.00067 (34±0.88)	0.10 ±0.0037 (7.9±0.22)	0.017 ±0.0010 (43±1.6)	27±0.32 (15±0.47)	1.5±0.22 (17±4.2)
120	52±5.2 (14±1.5)	23±2.8 (19±2.7)	0.017 ±0.00048 (29±1.1)	0.039 ±0.0066 (3.0±0.43)	0.0098 ±0.00080 (25±1.3)	22±1.5 (12±1.1)	1.3±0.066 (16±1.4)
140	37±0.34 (9.6±0.16)	9.7±0.53 (8.2±0.24)	0.011 ±0.0050 (20±0.80)	0.011 ±0.00093 (0.9±0.051)	0.0092 ±0.00055 (23±1.2)	18±0.81 (9.8±0.44)	0.95±0.19 (11±1.0)

160	23±0.14 (6.0±0.16)	9.4±0.33 (8.0±0.27)	0.0083 ±0.00025 (14±0.37)	0.0070 ±0.0033 (0.5±0.22)	0.0040 ±0.0021 (10±4.6	12±1.6 (6.7±0.82)	0.60 ±0.042 (7.2±1.0)
180	17±0.38 (4.5±0.18)	3.3±0.30 (2.8±0.15)	0.0043 ±0.00087 (7.5±1.5)	0.0055 ±0.00025 (0.4±0.015)	0.0018 ±0.00024 (4.5±0.54)	9.6±0.14 (5.3±0.10)	0.29 ±0.014 (3.5±0.46)
200	17±1.2 (4.4±0.24)	2.5±0.22 (2.1±0.24)	0.0039 ±0.000041 (6.9±0.0047)	0.0029 ±0.00054 (0.2±0.038)	0.00046 ±0.00029 (1.2±0.64)	2.6±0.46 (1.4±0.26)	0.17 ±0.0023 (2.0±0.20)

[a]The reaction was carried out in 50 mM Tris-HCl buffer (pH 8.0), 5 mM $MgCl_2$, 5.6 nM R18/D18 at 25ºC.

[b]Numbers in parentheses indicate values relative to the highest activity in each variant.
