# Supplementary material for: Val143 of human ribonuclease H2 is not critical for, but plays a role in determining catalytic activity and substrate specificity
Source: PLoS One. 2020 Feb 18;15(2):e0228774. doi: 10.1371/journal.pone.0228774 (PMC7028304; doi:10.1371/journal.pone.0228774)
Supplement: S4 Table — The original data of Fig 7 are shown. (PDF) [file pone.0228774.s009.pdf]

S4 Table. Data of CD spectra of Val143 variants.

nm	$[\theta]_{MRW}$ (deg•cm^2•dmol^{-1})						
	WT	V143I	V143G	V143D	V143K	V143Y	V143N
250	97	220	-10	17	67	-188	-84
249.8	34	298	29	22	53	-190	-70
249.6	1	258	37	-41	15	-83	-69
249.4	-21	229	6	-72	6	-60	-96
249.2	-134	145	34	-113	-64	-143	-154
249	-173	177	39	-85	-105	-204	-125
248.8	-181	125	30	-109	-134	-193	-156
248.6	-145	73	-79	-116	-195	-194	-191
248.4	-83	58	-54	-99	-89	-136	-222
248.2	-42	122	-106	-173	-40	-152	-211
248	-45	139	-100	-116	30	-129	-90
247.8	26	179	-63	-93	34	-91	-115
247.6	23	144	-151	-40	11	-87	-38
247.4	-9	42	-237	18	23	-47	-62
247.2	-59	54	-269	-79	15	-149	-72
247	-45	66	-222	-75	-32	-169	-142
246.8	2	89	-171	-122	-133	-154	-199
246.6	-7	128	-98	-180	-125	-169	-232
246.4	-75	-12	-74	-225	-205	-203	-289
246.2	-89	-13	-121	-177	-204	-245	-265
246	-103	-30	-71	-161	-185	-318	-256
245.8	-99	-89	-170	-74	-103	-320	-157
245.6	-118	43	-41	-134	-127	-259	-169
245.4	-159	35	-133	-68	-152	-233	-161
245.2	-249	73	-101	-97	-152	-261	-173
245	-241	-31	-165	-130	-139	-387	-233
244.8	-238	-64	-238	-216	-134	-395	-219
244.6	-253	-161	-353	-238	-196	-456	-255
244.4	-236	-175	-367	-216	-206	-358	-272
244.2	-231	-286	-358	-271	-335	-322	-362
244	-223	-209	-330	-352	-386	-307	-370

nm	WT	V143I	V143G	V143D	V143K	V143Y	V143N
243.8	-266	-220	-301	-353	-386	-363	-317
243.6	-185	-226	-369	-361	-404	-322	-319
243.4	-163	-138	-284	-352	-345	-311	-330
243.2	-150	-141	-256	-342	-378	-361	-397
243	-198	-86	-222	-442	-432	-352	-408
242.8	-298	-140	-293	-390	-481	-487	-405
242.6	-351	-296	-473	-520	-563	-457	-484
242.4	-387	-249	-509	-507	-572	-471	-486
242.2	-420	-300	-537	-538	-603	-399	-601
242	-482	-328	-627	-464	-566	-486	-542
241.8	-491	-460	-711	-483	-630	-520	-605
241.6	-468	-451	-786	-485	-631	-501	-619
241.4	-488	-380	-692	-545	-737	-529	-670
241.2	-536	-380	-739	-598	-788	-673	-698
241	-606	-459	-757	-565	-793	-778	-690
240.8	-660	-513	-829	-587	-745	-786	-699
240.6	-678	-620	-905	-618	-754	-729	-725
240.4	-686	-661	-947	-703	-790	-706	-755
240.2	-749	-736	-988	-740	-839	-792	-768
240	-693	-751	-891	-766	-897	-791	-765
239.8	-684	-697	-831	-844	-933	-882	-809
239.6	-648	-638	-870	-902	-1056	-730	-865
239.4	-662	-714	-950	-938	-1092	-829	-899
239.2	-742	-738	-982	-943	-1197	-832	-940
239	-806	-813	-962	-912	-1128	-932	-861
238.8	-920	-802	-904	-896	-1135	-936	-884
238.6	-919	-817	-912	-988	-1216	-913	-1051
238.4	-904	-853	-1022	-1020	-1223	-972	-1029
238.2	-887	-867	-1126	-1170	-1353	-1078	-1144
238	-967	-918	-1242	-1170	-1359	-1185	-1103
237.8	-1076	-945	-1239	-1234	-1483	-1222	-1238
237.6	-1059	-1073	-1370	-1281	-1566	-1186	-1330
237.4	-1164	-1165	-1505	-1377	-1565	-1264	-1382
237.2	-1200	-1139	-1563	-1461	-1585	-1324	-1404

nm	WT	V143I	V143G	V143D	V143K	V143Y	V143N
237	-1322	-1185	-1473	-1489	-1627	-1408	-1456
236.8	-1392	-1118	-1430	-1510	-1760	-1594	-1433
236.6	-1426	-1320	-1515	-1449	-1795	-1611	-1454
236.4	-1423	-1318	-1549	-1482	-1867	-1567	-1485
236.2	-1458	-1466	-1625	-1548	-1912	-1467	-1580
236	-1464	-1500	-1621	-1630	-1885	-1468	-1614
235.8	-1454	-1661	-1748	-1712	-1994	-1494	-1689
235.6	-1467	-1662	-1786	-1817	-2083	-1562	-1687
235.4	-1596	-1771	-1867	-1972	-2268	-1713	-1813
235.2	-1684	-1749	-1950	-2069	-2365	-1836	-1879
235	-1731	-1792	-2052	-2147	-2387	-1877	-1938
234.8	-1791	-1916	-2197	-2104	-2417	-1956	-2021
234.6	-1837	-1877	-2189	-2085	-2404	-1940	-1969
234.4	-1912	-1932	-2070	-2165	-2527	-1986	-2114
234.2	-1866	-1884	-2206	-2246	-2508	-1968	-2167
234	-1887	-1988	-2383	-2310	-2608	-1974	-2297
233.8	-1946	-2007	-2468	-2404	-2676	-2033	-2284
233.6	-2078	-2221	-2654	-2459	-2901	-2070	-2331
233.4	-2216	-2265	-2649	-2518	-2973	-2237	-2390
233.2	-2399	-2279	-2753	-2671	-3164	-2389	-2557
233	-2442	-2317	-2774	-2700	-3114	-2466	-2537
232.8	-2486	-2464	-2868	-2817	-3248	-2466	-2611
232.6	-2611	-2489	-2928	-2833	-3302	-2568	-2590
232.4	-2682	-2522	-3087	-2852	-3458	-2700	-2701
232.2	-2715	-2486	-3166	-2940	-3460	-2732	-2842
232	-2757	-2522	-3204	-3097	-3553	-2762	-2943
231.8	-2782	-2779	-3299	-3230	-3583	-2765	-2941
231.6	-2857	-2891	-3396	-3374	-3712	-2853	-2985
231.4	-2938	-3030	-3561	-3335	-3709	-3048	-2955
231.2	-3017	-3191	-3753	-3487	-3880	-3073	-3035
231	-3074	-3352	-3710	-3588	-4007	-3160	-3143
230.8	-3120	-3623	-3913	-3685	-4176	-3198	-3284
230.6	-3238	-3667	-3881	-3793	-4357	-3330	-3378
230.4	-3328	-3679	-4065	-3722	-4318	-3432	-3398

nm	WT	V143I	V143G	V143D	V143K	V143Y	V143N
230.2	-3425	-3628	-3967	-3834	-4453	-3482	-3479
230	-3487	-3719	-4087	-3904	-4568	-3561	-3635
229.8	-3517	-3963	-4211	-4040	-4709	-3615	-3882
229.6	-3598	-3855	-4323	-4090	-4735	-3753	-3894
229.4	-3673	-3980	-4543	-4146	-4744	-3832	-3878
229.2	-3806	-4109	-4660	-4297	-4907	-3945	-3927
229	-3885	-4311	-4902	-4269	-4949	-4030	-3955
228.8	-3985	-4482	-4917	-4381	-5095	-4131	-4142
228.6	-4119	-4630	-5074	-4567	-5209	-4236	-4244
228.4	-4230	-4652	-5078	-4694	-5364	-4293	-4229
228.2	-4360	-4519	-5290	-4886	-5533	-4392	-4358
228	-4402	-4538	-5351	-5023	-5671	-4412	-4472
227.8	-4459	-4668	-5422	-5159	-5810	-4543	-4577
227.6	-4494	-4890	-5472	-5358	-5996	-4501	-4695
227.4	-4610	-5036	-5463	-5480	-6177	-4687	-4754
227.2	-4760	-5159	-5640	-5638	-6344	-4905	-4897
227	-4860	-5351	-5850	-5594	-6337	-5044	-4986
226.8	-4941	-5445	-6063	-5565	-6417	-5021	-4928
226.6	-5088	-5481	-6111	-5640	-6472	-5091	-4981
226.4	-5156	-5542	-6247	-5639	-6539	-5195	-4953
226.2	-5212	-5652	-6300	-5775	-6589	-5378	-5191
226	-5256	-5843	-6627	-5848	-6739	-5356	-5288
225.8	-5311	-5836	-6769	-5955	-6870	-5411	-5449
225.6	-5476	-5919	-6903	-6076	-6966	-5528	-5511
225.4	-5639	-6001	-7065	-6075	-6927	-5638	-5512
225.2	-5729	-5913	-6828	-6162	-6956	-5590	-5561
225	-5729	-5931	-6794	-6235	-7168	-5559	-5676
224.8	-5727	-5891	-6616	-6331	-7480	-5548	-5948
224.6	-5776	-6052	-6664	-6345	-7663	-5772	-5901
224.4	-5914	-6266	-6957	-6574	-7819	-5876	-6010
224.2	-5978	-6250	-6971	-6571	-7816	-5998	-5923
224	-6050	-6272	-7134	-6679	-7856	-6008	-5978
223.8	-6076	-6272	-7157	-6723	-7881	-5953	-6013
223.6	-6098	-6372	-7133	-6794	-7825	-6095	-6069

nm	WT	V143I	V143G	V143D	V143K	V143Y	V143N
223.4	-6212	-6482	-7298	-6912	-7811	-6166	-6062
223.2	-6209	-6657	-7410	-7019	-7960	-6235	-6112
223	-6353	-6749	-7509	-7105	-8078	-6490	-6216
222.8	-6384	-6789	-7676	-7100	-8146	-6507	-6337
222.6	-6540	-6822	-7797	-7066	-8155	-6676	-6469
222.4	-6719	-6833	-8026	-6973	-8171	-6709	-6590
222.2	-6775	-6857	-7927	-7149	-8336	-6763	-6707
222	-6735	-6816	-7914	-7131	-8520	-6676	-6682
221.8	-6722	-6739	-7712	-7233	-8567	-6697	-6586
221.6	-6733	-6875	-7861	-7233	-8434	-6778	-6561
221.4	-6735	-7011	-7990	-7382	-8640	-6606	-6677
221.2	-6760	-7139	-8024	-7408	-8639	-6866	-6702
221	-6695	-7199	-8138	-7455	-8689	-6761	-6844
220.8	-6828	-7121	-8137	-7346	-8570	-6891	-6731
220.6	-6912	-7288	-8248	-7368	-8609	-6804	-6664
220.4	-7029	-7375	-8355	-7418	-8716	-6830	-6624
220.2	-7093	-7293	-8317	-7535	-8897	-6931	-6728
220	-7104	-7335	-8176	-7713	-8912	-6959	-6970
219.8	-7082	-7343	-8268	-7857	-8989	-6894	-7128
219.6	-7229	-7592	-8527	-7801	-9066	-6817	-7218
219.4	-7269	-7664	-8873	-7811	-9161	-6940	-7173
219.2	-7334	-7746	-8942	-7762	-9170	-6953	-7310
219	-7317	-7860	-8852	-7826	-8938	-6996	-7236
218.8	-7151	-7793	-8892	-7933	-9103	-7103	-7322
218.6	-7199	-7726	-8767	-8020	-9179	-7027	-7173
218.4	-7173	-7649	-8823	-8110	-9343	-7114	-7295
218.2	-7180	-7830	-8738	-8130	-9198	-6936	-7350
218	-7351	-7950	-8869	-8135	-9265	-6999	-7353
217.8	-7622	-7959	-9039	-8384	-9191	-7047	-7516
217.6	-7596	-7901	-9012	-8353	-9376	-7124	-7547
217.4	-7404	-7964	-8944	-8317	-9540	-7029	-7424
217.2	-7289	-7925	-8960	-8260	-9683	-7037	-7405
217	-7449	-8343	-9193	-8217	-9765	-7308	-7289
216.8	-7473	-8273	-9125	-8308	-9566	-7398	-7365

nm	WT	V143I	V143G	V143D	V143K	V143Y	V143N
216.6	-7649	-8178	-8839	-8073	-9337	-7257	-7338
216.4	-7655	-8032	-8673	-8140	-9169	-7131	-7388
216.2	-7739	-7971	-8908	-8108	-9259	-7085	-7376
216	-7639	-8078	-9213	-8229	-9532	-7315	-7526
215.8	-7626	-8091	-9359	-8240	-9646	-7480	-7538
215.6	-7699	-8188	-9111	-8346	-9744	-7517	-7521
215.4	-7794	-8014	-8999	-8249	-9719	-7633	-7275
215.2	-7885	-8053	-8714	-8248	-9756	-7585	-7501
215	-7732	-8132	-9027	-8190	-9768	-7808	-7512
214.8	-7793	-8149	-9015	-8388	-9745	-7810	-7954
214.6	-7878	-8292	-9223	-8395	-9775	-7834	-7885
214.4	-7835	-8136	-9116	-8402	-9608	-7579	-8094
214.2	-7740	-8147	-9042	-8433	-9653	-7363	-7812
214	-7786	-8080	-9088	-8476	-9669	-7346	-7747
213.8	-7877	-8105	-9208	-8556	-9750	-7444	-7630
213.6	-8026	-8213	-9362	-8700	-9833	-7699	-7626
213.4	-8122	-8563	-9695	-8729	-9866	-7862	-7772
213.2	-8033	-8650	-9727	-8869	-9933	-8174	-7838
213	-8108	-8823	-9837	-8684	-9637	-8047	-7909
212.8	-7914	-8649	-9717	-8744	-9483	-7845	-8071
212.6	-8053	-8776	-9680	-8827	-9477	-7686	-8028
212.4	-8235	-9184	-10092	-9009	-10009	-7606	-8084
212.2	-8237	-9179	-10262	-9142	-10336	-7843	-8176
212	-8320	-9266	-10170	-9137	-10289	-7951	-8235
211.8	-8136	-9132	-10008	-9104	-9970	-8165	-8254
211.6	-8277	-8799	-9858	-9077	-9969	-8327	-8252
211.4	-8405	-9023	-10178	-9311	-10456	-8685	-8416
211.2	-8310	-8889	-10416	-9293	-10963	-8349	-8550
211	-8044	-8884	-10305	-9407	-11515	-8414	-8707
210.8	-8439	-8922	-10498	-9465	-11381	-8233	-8563
210.6	-8598	-9039	-10527	-9679	-11182	-8905	-8591
210.4	-8840	-9251	-10763	-9615	-11114	-9266	-8619
210.2	-8834	-9571	-10408	-9688	-11274	-9555	-8731
210	-8748	-9492	-10129	-9803	-11122	-9178	-8842

nm	WT	V143I	V143G	V143D	V143K	V143Y	V143N
209.8	-8984	-9268	-10091	-10077	-11324	-8729	-9000
209.6	-9178	-8770	-10407	-10189	-11339	-8820	-9058
209.4	-9636	-9226	-10915	-10003	-11645	-8689	-9218
209.2	-9969	-9768	-11499	-9926	-11738	-8821	-9139
209	-9785	-10145	-11476	-10009	-11636	-8579	-9019
208.8	-9819	-10132	-11132	-10252	-11808	-8600	-9088
208.6	-9653	-9778	-10982	-10318	-12197	-9074	-8868
208.4	-10214	-9581	-11096	-10545	-11660	-9307	-9227
208.2	-10140	-10219	-11461	-10592	-11664	-9335	-9127
208	-9900	-10218	-11330	-10366	-11055	-9118	-9210
207.8	-9608	-10434	-11464	-10287	-11420	-9458	-9266
207.6	-9398	-10248	-11461	-10224	-11574	-9113	-9417
207.4	-9416	-9860	-11711	-10250	-11956	-9308	-9646
207.2	-9460	-9841	-11573	-10242	-12487	-8828	-9568
207	-9578	-9792	-10983	-10158	-12238	-8659	-9484
206.8	-9730	-9978	-11193	-10176	-11647	-8889	-9407
206.6	-9768	-9828	-11055	-9955	-11190	-8759	-8901
206.4	-9764	-9846	-11655	-9939	-10703	-8899	-8798
206.2	-9524	-9795	-11889	-10085	-10433	-9306	-8902
206	-9722	-9752	-11757	-10166	-10442	-9306	-9139
205.8	-9686	-9766	-11245	-10106	-10299	-9465	-9047
205.6	-9444	-9306	-10693	-9929	-10857	-8974	-8806
205.4	-8901	-9408	-10415	-9284	-11259	-8582	-8447
205.2	-9030	-9004	-10892	-9223	-10562	-8047	-8342
205	-8692	-8704	-11457	-9099	-10393	-7664	-8116
204.8	-8699	-8705	-10687	-9011	-10145	-7408	-7910
204.6	-8388	-8137	-10046	-8928	-10508	-7257	-7724
204.4	-7876	-8192	-9162	-8998	-9255	-7651	-7943
204.2	-7949	-7896	-9289	-8845	-8797	-7553	-7919
204	-7752	-7968	-9203	-8560	-7663	-6911	-7843
203.8	-7934	-7444	-8508	-8269	-7958	-6667	-7507
203.6	-7691	-6550	-7822	-7745	-8644	-6067	-6736
203.4	-7150	-5871	-7933	-7608	-7389	-6499	-6069
203.2	-6434	-5971	-7279	-7058	-6953	-6174	-5612

nm	WT	V143I	V143G	V143D	V143K	V143Y	V143N
203	-6022	-5687	-6878	-6280	-3475	-6188	-5045
202.8	-5754	-6007	-6472	-5659	-4459	-5658	-4870
202.6	-5422	-5430	-6399	-5033	-3629	-5381	-4534
202.4	-5165	-5334	-6477	-5323	-4596	-4498	-4505
202.2	-4465	-4980	-6667	-4805	-4032	-4277	-4643
202	-3700	-4368	-5680	-4877	-3100	-3985	-4893
201.8	-2918	-3471	-4920	-4013	-5309	-4238	-4397
201.6	-2287	-3332	-4734	-3932	-3837	-3411	-3812
201.4	-2669	-3436	-3140	-2994	-4248	-3210	-2717
201.2	-2805	-3100	-3027	-2530	-2909	-2247	-2222
201	-2342	-2408	-1614	-3032	86	-1052	-2110
200.8	-1884	-846	267	-2320	-1214	-341	-1789
200.6	-1367	-481	-86	-1894	1227	70	-1563
200.4	-343	-787	795	-646	-140	255	-1309
200.2	110	-103	698	76	-2404	-486	-299
200	326	221	1665	-353	-1232	-340	-206
