# Supplementary material for: Val143 of human ribonuclease H2 is not critical for, but plays a role in determining catalytic activity and substrate specificity
Source: PLoS One. 2020 Feb 18;15(2):e0228774. doi: 10.1371/journal.pone.0228774 (PMC7028304; doi:10.1371/journal.pone.0228774)
Supplement: S5 Table — The original data of Fig 8 are shown. (PDF) [file pone.0228774.s010.pdf]

S5 Table. Dependence of fraction unfolded of Val143 variants on the temperature.

Temp.	Fraction unfolded						
(ºC)	WT	V143G	V143I	V143D	V143K	V143Y	V143N
30	N.D.[a]	1	-2	-4	3	0	2
30.1	N.D.	3	-9	-1	2	1	0
30.2	-2	-1	-3	-4	2	3	-2
30.3	-7	-5	-4	1	-1	-4	-3
30.4	2	-1	1	-1	5	-2	0
30.5	4	-5	1	0	-2	6	-3
30.6	-1	2	2	-2	2	-3	-2
30.7	0	0	-1	-2	8	-6	1
30.8	3	4	-6	-3	-5	-2	1
30.9	-2	-1	-5	-2	1	-7	3
31	0	-3	1	-3	1	-7	-4
31.1	3	-6	-3	-1	-2	-4	2
31.2	0	-4	0	3	-1	-4	1
31.3	2	0	-4	-3	-2	-6	-2
31.4	1	1	-1	-1	0	-1	2
31.5	0	-3	-3	-2	2	1	-2
31.6	3	-2	-4	-2	1	-3	-4
31.7	-3	-1	-2	-1	-2	4	0
31.8	0	-2	-1	2	4	-6	0
31.9	1	0	2	1	-2	-2	-4
32	-1	-1	1	-1	3	-4	-1
32.1	-3	3	-5	0	3	0	2
32.2	4	2	-3	-2	3	-1	3
32.3	-3	-5	-4	-3	5	7	2
32.4	2	-4	-4	-1	3	-1	-4
32.5	-6	-1	-1	-2	6	-9	-1
32.6	4	1	1	-1	8	0	-3
32.7	6	-6	2	3	0	1	1
32.8	1	4	-2	2	-1	2	-1
32.9	1	0	-2	-3	-2	-2	-1
33	1	3	2	1	-2	-6	-3
33.1	-5	3	-1	-1	1	4	0

Temp.	WT	V143G	V143I	V143D	V143K	V143Y	V143N
33.2	4	2	-3	1	1	-1	1
33.3	3	2	2	2	3	9	-4
33.4	3	-1	4	1	0	-5	-3
33.5	2	-5	5	3	3	-4	-4
33.6	5	-5	1	-2	4	-9	3
33.7	2	-1	6	-1	-1	-1	-2
33.8	-4	-2	0	2	-1	6	-1
33.9	6	-1	-1	5	1	4	-1
34	3	-2	1	5	-2	0	0
34.1	6	2	-4	2	0	5	-5
34.2	7	0	-3	1	0	0	-3
34.3	-3	-1	-1	2	-1	-4	3
34.4	0	4	2	2	4	-7	-1
34.5	3	0	3	0	0	-5	-1
34.6	2	-1	2	1	1	-4	0
34.7	-5	-3	2	4	2	-6	-4
34.8	-3	-4	-2	-2	0	-5	4
34.9	8	-2	-3	-4	7	9	1
35	0	0	-4	-1	2	2	-3
35.1	6	5	2	-2	3	3	0
35.2	5	0	5	-1	0	5	1
35.3	1	5	4	0	-2	-5	0
35.4	-2	2	-2	4	3	-5	-3
35.5	-1	-5	-1	-1	3	-3	0
35.6	-2	0	-1	-1	-1	-8	1
35.7	8	-3	-1	-1	2	-1	2
35.8	0	-4	-4	-2	2	0	-2
35.9	6	-5	0	0	6	-2	2
36	-4	-2	3	0	6	4	1
36.1	1	1	3	-4	-1	-4	1
36.2	0	6	1	0	1	-6	1
36.3	0	-2	1	-1	3	-4	3
36.4	-6	-8	5	2	4	0	0
36.5	5	-1	0	1	6	4	0
36.6	-1	2	5	-3	4	0	4

Temp.	WT	V143G	V143I	V143D	V143K	V143Y	V143N
36.7	4	1	0	-4	4	3	0
36.8	8	-3	1	-3	6	5	-3
36.9	6	-2	1	2	5	-2	1
37	9	3	-2	1	3	3	3
37.1	4	5	-1	0	3	1	-1
37.2	7	3	5	4	9	5	0
37.3	-1	2	0	-4	7	-2	2
37.4	1	2	1	0	5	2	4
37.5	3	1	-1	1	1	1	1
37.6	1	1	-1	1	3	-10	1
37.7	8	0	-1	0	4	-4	7
37.8	4	4	5	3	3	1	2
37.9	2	-2	0	1	3	-3	4
38	-2	0	2	3	-1	-3	3
38.1	0	-3	3	2	0	5	0
38.2	-1	2	0	3	-1	0	7
38.3	-4	1	-3	0	2	-2	2
38.4	-1	1	8	-1	6	2	-3
38.5	7	-3	8	-3	-1	11	6
38.6	5	3	0	0	3	-2	8
38.7	1	4	2	2	8	-7	4
38.8	1	1	-1	5	6	-6	2
38.9	1	-3	6	4	5	4	0
39	1	-1	7	3	1	5	1
39.1	2	3	6	4	8	-6	2
39.2	3	4	3	-1	8	1	1
39.3	8	2	4	3	0	-1	6
39.4	3	-2	2	4	6	4	1
39.5	1	0	3	1	1	4	3
39.6	3	1	2	1	2	-1	5
39.7	4	1	1	2	8	-5	2
39.8	5	-4	4	3	11	-1	5
39.9	7	0	2	3	9	0	5
40	-2	-3	3	-1	2	-1	6
40.1	5	-2	8	7	5	-2	5

Temp.	WT	V143G	V143I	V143D	V143K	V143Y	V143N
40.2	4	3	4	4	6	1	3
40.3	5	0	3	7	-1	2	4
40.4	4	-3	5	0	4	1	2
40.5	7	-3	2	5	6	-4	5
40.6	3	1	3	5	4	6	4
40.7	7	6	3	6	5	4	2
40.8	0	4	4	6	7	5	7
40.9	5	5	1	1	4	1	2
41	-2	4	6	3	7	0	3
41.1	2	2	5	1	3	3	8
41.2	6	2	3	4	6	-3	3
41.3	10	2	6	7	10	1	4
41.4	3	3	3	1	11	2	5
41.5	1	-4	5	3	7	1	7
41.6	-3	3	5	6	12	4	4
41.7	6	4	5	4	2	-3	8
41.8	7	0	1	4	4	-2	3
41.9	5	1	8	6	4	9	4
42	7	0	3	5	3	3	2
42.1	1	0	6	4	9	0	1
42.2	13	3	2	4	5	1	7
42.3	3	-1	6	6	4	2	5
42.4	9	3	6	3	9	-1	1
42.5	-1	-3	3	4	11	4	2
42.6	2	1	6	4	4	5	5
42.7	-2	1	7	2	9	-3	2
42.8	5	5	8	6	8	-4	3
42.9	4	5	4	6	6	1	5
43	3	0	9	5	12	0	8
43.1	3	0	10	7	3	8	8
43.2	10	-1	6	5	6	7	7
43.3	10	3	7	6	5	9	0
43.4	0	1	8	3	8	1	0
43.5	0	-1	5	6	6	1	4
43.6	1	6	9	7	10	-4	5

Temp.	WT	V143G	V143I	V143D	V143K	V143Y	V143N
43.7	7	-2	7	4	7	-3	10
43.8	9	0	10	5	7	-1	6
43.9	6	-2	6	8	9	5	4
44	14	1	2	3	7	3	4
44.1	11	1	3	7	10	4	8
44.2	0	1	4	5	10	-2	7
44.3	3	2	4	4	10	-1	10
44.4	6	7	9	4	11	4	9
44.5	10	4	7	3	8	2	10
44.6	2	4	5	4	11	5	10
44.7	12	1	6	5	13	1	10
44.8	9	3	8	10	9	2	17
44.9	6	-2	6	8	10	6	10
45	4	2	8	9	12	0	10
45.1	7	1	10	8	6	-1	9
45.2	1	3	10	6	10	6	9
45.3	6	-2	11	8	9	3	8
45.4	2	6	8	5	14	-1	8
45.5	1	1	10	8	9	9	8
45.6	3	2	9	9	7	4	8
45.7	2	2	10	10	9	5	10
45.8	12	3	12	8	10	5	4
45.9	0	2	6	6	13	4	14
46	0	0	5	4	15	10	9
46.1	6	4	2	8	11	7	9
46.2	10	2	9	4	10	6	9
46.3	7	5	10	11	5	5	7
46.4	6	-1	8	8	12	13	11
46.5	1	8	10	11	14	-1	11
46.6	0	2	12	8	10	3	14
46.7	5	4	14	10	12	12	11
46.8	4	3	10	9	14	8	11
46.9	2	-1	7	11	11	4	7
47	7	2	3	13	14	1	10
47.1	8	3	9	8	16	0	12

Temp.	WT	V143G	V143I	V143D	V143K	V143Y	V143N
47.2	9	1	12	12	14	9	13
47.3	3	3	11	12	15	4	13
47.4	3	8	11	12	13	2	11
47.5	6	6	15	12	14	0	11
47.6	12	6	9	9	16	6	12
47.7	5	1	14	13	19	7	13
47.8	9	5	15	12	16	12	14
47.9	7	5	15	14	10	9	13
48	11	7	14	13	17	8	11
48.1	11	3	16	15	20	8	9
48.2	3	3	15	9	17	17	10
48.3	12	8	15	14	13	7	11
48.4	12	7	15	12	14	6	5
48.5	4	5	17	9	15	7	12
48.6	10	7	16	14	22	12	12
48.7	7	5	16	14	24	12	14
48.8	7	2	16	15	17	19	13
48.9	12	5	14	14	18	16	14
49	13	4	17	12	23	16	14
49.1	12	6	20	14	23	17	17
49.2	8	4	16	15	19	13	14
49.3	15	7	24	17	17	13	15
49.4	14	8	21	18	19	12	13
49.5	8	5	16	18	18	11	12
49.6	12	5	19	20	21	16	18
49.7	10	14	16	16	22	12	16
49.8	12	5	26	17	16	12	16
49.9	20	6	17	13	21	20	18
50	14	8	23	15	22	13	20
50.1	13	3	22	17	21	5	19
50.2	15	9	21	20	22	13	17
50.3	14	4	21	18	16	13	19
50.4	11	14	22	24	17	15	19
50.5	13	6	20	22	21	18	17
50.6	20	7	23	18	19	13	19

Temp.	WT	V143G	V143I	V143D	V143K	V143Y	V143N
50.7	13	11	20	20	18	21	16
50.8	14	9	22	20	18	23	20
50.9	17	8	28	18	22	16	23
51	17	9	23	20	24	20	25
51.1	17	12	24	24	19	18	24
51.2	14	8	22	19	22	20	25
51.3	15	8	26	24	24	19	19
51.4	19	10	25	22	23	21	20
51.5	23	10	26	25	22	29	22
51.6	21	13	29	26	26	15	21
51.7	25	14	31	21	29	20	28
51.8	24	16	28	25	27	20	24
51.9	23	12	26	25	28	23	22
52	20	19	31	30	27	21	23
52.1	20	17	31	25	28	29	25
52.2	19	13	26	25	29	22	27
52.3	27	17	31	24	25	24	21
52.4	24	17	38	25	26	25	25
52.5	22	18	35	28	27	20	26
52.6	26	17	41	31	26	25	28
52.7	20	18	38	25	31	23	30
52.8	22	15	35	28	30	28	26
52.9	22	17	44	26	31	25	27
53	21	21	37	29	32	25	29
53.1	19	19	41	33	36	28	33
53.2	21	22	42	32	38	27	29
53.3	21	23	40	36	37	33	30
53.4	27	20	41	33	34	30	32
53.5	39	21	39	30	33	28	33
53.6	26	22	40	34	37	32	32
53.7	26	23	41	39	36	39	36
53.8	33	21	47	36	34	37	36
53.9	35	29	46	33	36	36	38
54	37	30	46	36	37	33	37
54.1	31	27	47	35	40	39	44

Temp.	WT	V143G	V143I	V143D	V143K	V143Y	V143N
54.2	35	32	51	36	40	39	38
54.3	44	32	53	44	36	42	37
54.4	37	39	51	41	42	46	44
54.5	40	39	56	40	36	48	43
54.6	38	33	55	40	38	46	41
54.7	40	30	57	41	40	48	36
54.8	40	36	58	45	41	47	46
54.9	47	36	60	43	43	42	42
55	46	40	56	44	45	47	43
55.1	48	41	59	43	47	46	49
55.2	47	41	60	48	48	53	55
55.3	44	43	71	49	48	53	52
55.4	56	45	72	46	50	44	51
55.5	46	43	68	48	50	41	54
55.6	52	46	70	55	53	58	50
55.7	46	48	68	51	51	56	58
55.8	47	56	69	57	50	52	53
55.9	58	52	73	52	56	59	57
56	64	57	64	59	51	60	64
56.1	55	53	75	59	54	59	62
56.2	62	68	80	56	51	68	64
56.3	59	68	77	60	56	75	62
56.4	67	60	78	64	61	72	62
56.5	70	60	78	63	60	72	68
56.6	57	65	88	60	59	76	64
56.7	63	67	77	60	62	74	74
56.8	68	75	90	66	67	77	73
56.9	73	76	88	71	67	72	70
57	71	83	86	68	70	79	74
57.1	71	80	90	69	73	76	75
57.2	68	73	86	75	70	74	74
57.3	74	75	84	73	70	76	80
57.4	69	81	85	71	73	72	75
57.5	72	76	89	71	76	82	76
57.6	75	75	96	78	79	78	76

Temp.	WT	V143G	V143I	V143D	V143K	V143Y	V143N
57.7	89	80	93	82	75	82	84
57.8	80	88	95	78	76	80	86
57.9	81	86	94	82	87	88	87
58	89	75	96	81	83	79	86
58.1	89	83	92	82	78	88	89
58.2	85	85	96	81	79	88	89
58.3	84	81	91	81	84	93	85
58.4	86	93	91	91	86	91	85
58.5	93	92	97	88	80	85	92
58.6	84	89	92	85	82	88	96
58.7	87	92	93	86	87	92	95
58.8	89	91	95	91	87	95	91
58.9	86	95	96	88	98	95	92
59	94	97	95	90	90	96	93
59.1	100	101	95	88	90	99	92
59.2	98	89	101	87	85	85	93
59.3	91	102	101	94	91	89	96
59.4	95	99	98	96	92	93	99
59.5	89	92	101	88	91	88	96
59.6	98	96	98	91	92	102	100
59.7	98	103	94	92	90	94	90
59.8	92	99	97	92	93	100	97
59.9	93	96	102	96	94	96	97
60	98	96	100	91	98	94	96
60.1	102	99	96	98	98	93	100
60.2	99	99	102	97	92	101	101
60.3	100	92	103	97	95	102	102
60.4	96	101	100	97	94	101	104
60.5	91	96	103	96	94	104	94
60.6	97	98	99	95	94	91	95
60.7	99	102	99	95	96	103	96
60.8	95	99	100	93	94	96	97
60.9	99	104	102	89	95	106	98
61	97	100	98	96	98	103	101
61.1	97	98	101	95	96	100	100

Temp.	WT	V143G	V143I	V143D	V143K	V143Y	V143N
61.2	93	101	104	99	97	96	96
61.3	92	106	98	101	103	102	100
61.4	98	104	101	102	102	92	99
61.5	94	105	97	98	96	103	100
61.6	95	97	99	96	97	106	97
61.7	98	88	98	94	98	101	100
61.8	99	99	101	96	101	100	104
61.9	97	102	102	96	104	91	97
62	95	103	104	98	99	93	98
62.1	94	96	101	102	105	110	102
62.2	95	93	106	100	104	101	106
62.3	99	92	100	99	102	95	97
62.4	100	102	95	99	100	95	102
62.5	102	101	96	100	102	100	103
62.6	101	98	98	100	100	97	97
62.7	98	99	98	99	100	104	103
62.8	100	102	96	101	97	104	99
62.9	104	106	100	103	96	99	99
63	98	107	96	102	100	100	100
63.1	100	97	101	102	103	99	106
63.2	90	99	101	101	103	94	103
63.3	94	99	97	100	99	98	100
63.4	96	103	96	100	103	97	98
63.5	97	95	98	98	106	97	98
63.6	93	99	102	98	109	97	100
63.7	91	95	102	98	104	102	99
63.8	99	100	100	94	102	112	101
63.9	98	106	102	103	101	104	103
64	102	103	96	100	98	95	105
64.1	103	106	96	97	102	94	102
64.2	95	96	103	104	97	104	101
64.3	104	94	98	102	99	103	97
64.4	110	102	100	102	100	101	104
64.5	99	95	102	100	109	103	96
64.6	98	101	102	101	103	105	100

Temp.	WT	V143G	V143I	V143D	V143K	V143Y	V143N
64.7	104	101	97	103	109	112	102
64.8	101	104	105	103	99	106	99
64.9	100	102	98	101	100	101	101
65	106	97	100	99	101	106	98
65.1	101	102	102	97	94	106	97
65.2	102	106	101	98	100	98	97
65.3	103	105	100	102	100	103	96
65.4	97	102	101	99	101	95	99
65.5	98	100	104	98	100	103	98
65.6	100	99	100	101	94	98	101
65.7	103	96	103	106	99	102	103
65.8	99	105	105	100	103	97	101
65.9	96	97	99	100	96	96	99
66	104	100	102	103	96	98	101
66.1	105	109	103	99	98	105	100
66.2	94	109	99	99	104	99	104
66.3	99	101	101	103	101	96	97
66.4	96	100	93	100	101	105	97
66.5	100	106	99	102	105	102	97
66.6	102	99	100	103	106	108	101
66.7	97	102	100	101	100	102	99
66.8	96	99	101	99	103	94	102
66.9	105	104	97	104	96	100	101
67	100	99	103	100	98	103	101
67.1	98	99	103	103	101	108	108
67.2	108	105	99	105	99	103	98
67.3	94	104	98	105	105	100	100
67.4	98	97	99	103	99	100	100
67.5	98	95	100	102	98	104	100
67.6	99	99	102	104	104	97	101
67.7	93	100	99	104	104	98	100
67.8	107	101	106	101	102	105	99
67.9	100	108	99	103	97	96	100
68	98	100	102	105	101	100	99
68.1	95	101	104	99	100	100	101

Temp.	WT	V143G	V143I	V143D	V143K	V143Y	V143N
68.2	97	108	102	106	99	104	101
68.3	106	104	103	103	105	108	105
68.4	107	97	102	92	95	105	100
68.5	97	101	101	92	99	99	101
68.6	94	99	102	96	98	98	103
68.7	100	102	100	103	102	101	100
68.8	102	105	99	102	102	106	104
68.9	98	98	101	99	104	109	107
69	102	106	95	103	100	99	105
69.1	94	103	96	103	97	96	100
69.2	101	102	99	103	99	103	99
69.3	105	111	96	102	101	96	104
69.4	102	104	101	101	101	105	103
69.5	97	97	102	103	100	103	101
69.6	97	99	98	104	100	102	98
69.7	102	101	106	102	103	102	101
69.8	96	103	106	99	102	102	101
69.9	111	102	103	102	98	103	97
70	98	103	101	100	100	101	98

[a]N.D. indicates Not Detected.
